# Identification of HIV Rapid Mutations Using Differences in Nucleotide Distribution over Time

**DOI:** 10.3390/genes13020170

**Published:** 2022-01-19

**Authors:** Nan Sun, Jie Yang, Stephen S.-T. Yau

**Affiliations:** 1 Department of Mathematical Sciences, Tsinghua University, Beijing 100084, China; sunn19@mails.tsinghua.edu.cn; 2 Department of Mathematics, Statistics, and Computer Science, University of Illinois at Chicago, Chicago, IL 60607, USA; jyang06@uic.edu; 3 Yanqi Lake Beijing Institute of Mathematical Sciences and Applications, Beijing 101408, China

**Keywords:** mutations, natural vector, human immunodeficiency virus, nucleotide distribution difference

## Abstract

Mutation is the driving force of species evolution, which may change the genetic information of organisms and obtain selective competitive advantages to adapt to environmental changes. It may change the structure or function of translated proteins, and cause abnormal cell operation, a variety of diseases and even cancer. Therefore, it is particularly important to identify gene regions with high mutations. Mutations will cause changes in nucleotide distribution, which can be characterized by natural vectors globally. Based on natural vectors, we propose a mathematical formula for measuring the difference in nucleotide distribution over time to investigate the mutations of human immunodeficiency virus. The studied dataset is from public databases and includes gene sequences from twenty HIV-infected patients. The results show that the mutation rate of the nine major genes or gene segment regions in the genome exhibits discrepancy during the infected period, and the *Env* gene has the fastest mutation rate. We deduce that the peak of virus mutation has a close temporal relationship with viral divergence and diversity. The mutation study of HIV is of great significance to clinical diagnosis and drug design.

## 1. Introduction

Human immunodeficiency virus (HIV) is a highly variable virus and has a high replication rate in participants, which leads to many quite different genetic variations of the viruses [1]. The HIV-1 polymerase is very error-prone, with the net result to generate escape mutations on newly generated viruses (evolution) undetected by the immune system [2]. The two types of HIV—HIV-1 and HIV-2—will cause acquired immunodeficiency syndrome (AIDS) [3], but HIV-1 is more virulent and infective than HIV-2 [4]. It is the cause of most HIV infections globally, and thousands of infected people appear every year [5]. HIV-1 is composed of two copies of positive-sense single-stranded RNA, and the genome length is about 9.2–9.8 kb (RefSeq accession number in GenBank is NC_001802, sequence length is 9181 bp). There are nine major genes, including three structural genes—*Gag*, *Pol*, and *Env*; two regulatory genes—*Tat* and *Rev*; and four auxiliary genes—*Nef*, *Vpr*, *Vpu*, and *Vif*. The mutation rate of each gene in the HIV-1 genome sequence is different, and the *Env* gene mutation rate is the highest [6]. The mutation rate detection of the gene segment or whole genome has important guiding significance for HIV monitoring, diagnosis, vaccine, and drug treatment.

CD4 count and viral load matter a great deal to measure patients’ disease progression. HIV mainly attacks CD4+ T cells [7], macrophages and dendritic cells [8] using the CD4 receptor as a docking site [9]. The infection of HIV results in a gradual change in the number of CD4-expressing T cells [10], which can be measured by CD4 count. Virus load is another important factor to assess the immune system. At the acute infection stage, the virus rapidly propagates, the virus content in each milliliter of blood can reach millions, and CD4 count will also decrease significantly. After the stage of acute HIV infection, the strong response of the immune system inhibits the activity of the virus and reduces the amount of virus in the blood. The length of incubation period is affected by many factors, ranging from 3 years to 20 years [11,12]. Usually, once the number of CD4 per microliter of blood is less than 200 [13], this means that the immune system is almost compromised and the human body can no longer effectively deal with many common infections. Patients will suffer from AIDS and soon die of cancer [14,15]. Therefore, the relationship of virus mutation, viral load and CD4 count is of great importance.

Mutations may create new HIV quasi-species, so comparison of the mutational nucleotide sequence with the original sequence is necessary. Methods to compare sequences include alignment and alignment-free methods. Alignment methods contain pairwise or multiple sequence alignment [16,17]. Alignment-free methods include power spectrum [18,19,20], k-mer theory [21], the density-based method [22], and the natural vector method [23], among others [24]. Natural vector (NV) was proposed to classify viral genomic sequences by Yau’s team in 2013 [23], and has been successfully applied to many studies [25,26,27]. Natural vector characterizes the distribution of four nucleotides in the genome, including their counts, mean positions and second central moments of location. Mutations will lead to changes in nucleotide distribution, which inspires us to use the difference in natural vector over time to measure mutations globally.

In this paper, we present a mathematical formula of nucleotide distribution difference over time to investigate the mutations. This is defined as the difference in natural vectors of the two nucleotide sequences divided by the corresponding difference in time points. We use the equation to explore the gene mutations of HIV. The studied dataset includes gene sequences from twenty HIV-1-infected patients. The results show that the mutation rate of the main genes or segment regions in the genome exhibits discrepancy during the infected period. There is a consistent pattern in the temporal sense among the mutation rate of *Env* gene sequences, viral divergence, and viral diversity. This study provides a meaningful and advanced tool to study mutations.

## 2. Materials and Methods

### 2.1. The Traditional 12-Dimensional Natural Vector

Natural vector is a powerful method to characterize the statistical features of biological sequences. The definition is as follows. Suppose the genomic sequence is $S=s_{1}\;s_{2}\;s_{3}\ldots s_{n}$ with length n. For $k\in L=\left\{A,C,G,T/U\right\}$, the indicator function is

(1) $$w_{k}\left(s_{i}\right)=\left\{\begin{array}{c} 1,\;\;\;\;\;\;if\;s_{i}=k\;\\ 0,\;\;\;\;otherwise \end{array}\right.,\;$$

where $s_{i}\in L,i=1,2,3,\ldots ,n$. Then, the distribution of four nucleotides can be described by a 12-dimensional natural vector:

(2) $$\left(n_{A},\;n_{C},n_{G},n_{T},\mu_{A},\mu_{C},\mu_{G},\mu_{T},D_{2}^{A},D_{2}^{C},D_{2}^{G},D_{2}^{T}\right),\;$$

$n_{k}$ denotes the count of nucleotide *k* within sequence *S*:

(3) $$n_{k}=\overset{n}{\underset{i=1}{\sum }}w_{k}\left(s_{i}\right),\;$$

$\mu_{k}$ specifies the average location of nucleotide *k* within sequence *S*:

(4) $$\mu_{k}=\overset{n}{\underset{i=1}{\sum }}i\frac{w_{k}\left(s_{i}\right)}{n_{k}},\;$$

$D_{2}^{k}$ is the second central moment of positions of nucleotide *k* within sequence *S*:

(5) $$D_{2}^{k}=\overset{n}{\underset{i=1}{\sum }}\frac{{\left(i-\mu_{k}\right)}^{2}w_{k}\left(s_{i}\right)}{n_{k}n},\;$$

Here, we give an example how to calculate the vector. If the sequence is ACTGCTATGA, the indicator functions are $w_{A}=1000001001$, $w_{C}=0100100000$, $w_{G}=0001000010$, and $w_{T}=0010010100$. Each component of the vector is as follows:

- $n_{A}=3,\;n_{C}=2,\;n_{G}=2,\;n_{T}=3$
- $\mu_{A}=\frac{1+7+10}{3}=6,\;\mu_{C}=\frac{2+5}{2}=\frac{7}{2},\;\mu_{G}=\frac{4+9}{2}=\frac{13}{2},\;\mu_{T}=\frac{3+6+8}{3}=\frac{17}{3}$.
- $D_{2}^{A}=\frac{{\left(1-6\right)}^{2}+{\left(7-6\right)}^{2}+{\left(10-6\right)}^{2}}{3\cdot 10}=\frac{21}{15},\;D_{2}^{C}=\frac{{\left(2-\frac{7}{2}\right)}^{2}+{\left(5-\frac{7}{2}\right)}^{2}}{2\cdot 10}=\frac{9}{40},\;$
  $D_{2}^{G}=\frac{{\left(4-\frac{13}{2}\right)}^{2}+{\left(9-\frac{13}{2}\right)}^{2}}{2\cdot 10}=\frac{5}{8},\;D_{2}^{T}=\frac{{\left(3-\frac{17}{3}\right)}^{2}+{\left(6-\frac{17}{3}\right)}^{2}+{\left(8-\frac{17}{3}\right)}^{2}}{3\cdot 10}=\frac{19}{45}$.

Then, the 12-dimensional natural vector is:

$$\left(3,\;2,\;2,\;3,\;6,\frac{7}{2},\;\frac{13}{2},\frac{17}{3},\frac{21}{15},\frac{9}{40},\frac{5}{8},\frac{19}{45}\right).$$

### 2.2. Measure the Changes of Nucleotide Distribution over Time Based on Natural Vector

Sequence mutations may change the genetic information of organisms and obtain selective competitive advantages through natural selection to adapt to environmental changes. This is the driving force of species evolution. All life activities of organisms are related to proteins. Gene mutations may change the structure or function of translated proteins, which may result in abnormal cell operation, a variety of diseases and even cancer [1]. Therefore, it is particularly important to identify gene regions with high mutations.

Counting the number and type is an important method to detect mutations. Mutations will cause changes in nucleotide distribution. Another intuitive idea is to use the change in natural vector over time to measure the mutations globally. Suppose sequence $S_{1}$ is examined at time point $D_{1}$; it mutates over a period of time and becomes sequence $S_{2}$ at time point $D_{2}$. Both sequences are transformed into the corresponding natural vectors $NV_{1}$ and $NV_{2}$ first. Then, the nucleotide distribution difference over time (NDDT) of the two sequences is described as:

(6) $$\mathrm{NDDT}=\frac{{\left|\left|NV_{2}-NV_{1}\right|\right|}_{2}}{D_{2}-D_{1}}.\;$$

where ${\left|\left|\cdot \right|\right|}_{2}$ is the $l_{2}-norm$, which has commonly been used in previous NV studies. We use the formula to measure the HIV mutation rates and will verify the rationality of the definition in the results part.

### 2.3. Validation Dataset

The first longitudinal sequence dataset covers the whole genomes of eleven HIV-1-infected patients without therapy in Sweden, with long-term follow-up from 1990 to 2003 [28] (https://hiv.biozentrum.unibas.ch, accessed on 27 June 2021). These data were sampled with 5–12 time points. Patient 10 was removed from the dataset due to a shortage of time points. Besides the complete genome of each patient, clinical data, including CD4 count, viral load and the position of each gene in the genome sequence, are available. Ten patients’ information is presented in Supplementary Dataset 1.

The second longitudinal sequence dataset includes 1337 sequences of a patient at 17 time points during the first three years of infection [29,30,31] (https://www.hiv.lanl.gov/content/sequence/HIV/SI_alignments/set10.html, accessed on 25 August 2021). The sequences belong to eight sequence segment regions: *Nef*, p17, p24, RT, *Vpu*, gp41, *Vpr*, and *Tat*. Detailed information can be found in Supplementary Dataset 2.

The third longitudinal dataset includes 1032 *Env* gene sequences, with an average of 12 sampling time points per person (https://www.hiv.lanl.gov/content/sequence/HIV/SI_alignments/datasets.html, accessed on 27 June 2021). The C2-V5 region of *Env* is used for research because it plays an important role in encoding the target of immune responses and shows a high degree of variation [6,32,33]. The sequences belong to nine HIV-positive participants, who were tracked over a 6~12 years period starting at the time of seroconversion. These participants were all homosexual men enrolled in the Multicenter AIDS Cohort Study (MACS, http://aidscohortstudy.org, accessed on 27 June 2021) [34]. The statistical information of the sampling time points and the *Env* gene sequence number of these 1032 sequences are shown in Supplementary Dataset 3. The virus has been evolving constantly in each infected patient. There might exist different virus variations at the same time point, and more than one sequence was obtained at a given time point. Seven men were treated during this study. Participant 8 did not take any antiretroviral therapy during the whole study. Participant 11 was the slowest progressor and did not receive treatment either. Participant 9 was another typical non-progressor but progressed subsequently [6].

## 3. Results

### 3.1. NDDT Comparison of the Nine Major Genes in HIV-1 Genome

The exploration of nucleotide distribution difference over time of the nine main genes of the HIV-1 genome—*Gag*, *Pol*, *Env*, *Tat*, *Rev*, *Nef*, *Vpr*, *Vpu*, and *Vif*—is of great significance. Note that most genes are composed of multiple sequence segments in dataset 1: *Env* includes gp120 and gp41; *Gag* includes P17, P24, P2, P7, P1 and P6; *Pol* includes PR, RT, P15 and IN; *Tat* and *Rev* consist of two segments, respectively; and the remaining four genes—*Vif*, *Vpr*, *Vpu*, and *Nef*—only have one segment, respectively.

**Figure 1:**
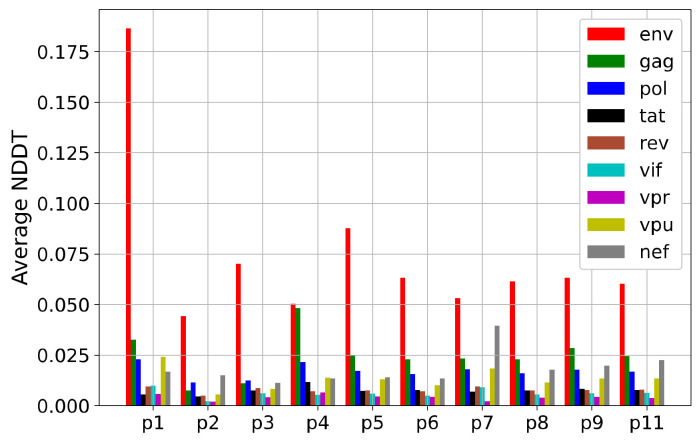
The average nucleotide distribution difference of the nine genes for each patient over all time periods.

We first parse each segment sequence from the genome according to their position records and assemble the segments of each gene together; we then transform each gene sequence into a 12-dimensional natural vector. We calculate the NDDTs of the nine genes for each patient, and show the results in Figures S1–S10. The horizontal axis represents the time from infection, and the red dot represents the nucleotide distribution difference of *Env* gene over time. The figures show that the mutation rate of the *Env* gene is the highest among almost all time periods. To better describe this fact, we take the average of the mutation rates of the nine genes during the whole study period and exhibit the results in Figure 1. Red bars indicate the mutation rate of *Env*, which is the highest compared with those of other genes. *Gag*, *Pol*, *Nef*, and *Vpu* also show high mutation rates.

**Figure 2:**
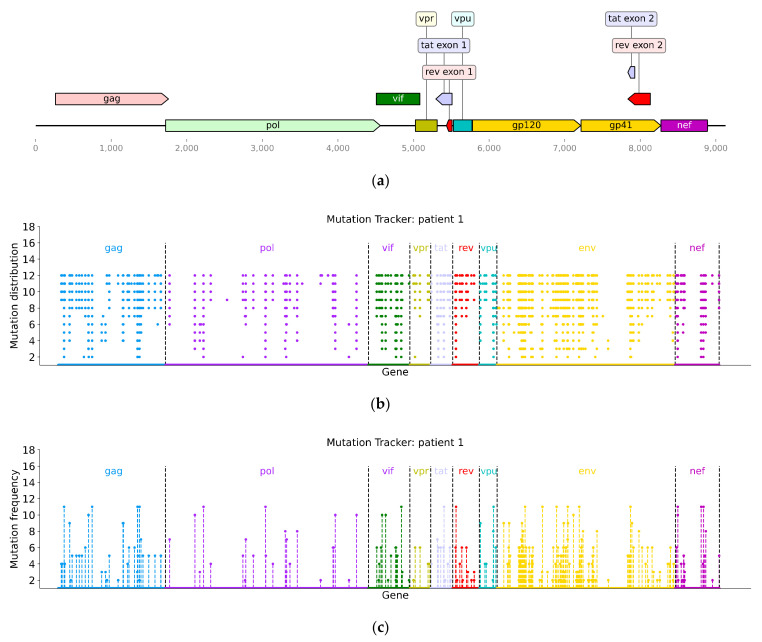
(**a**) Structure of the RNA genome of HIV-1. The structure is drawn through the DNA viewer (https://pypi.org/project/dna-features-viewer/, accessed on 17 April 2021); (**b**) Single nucleotide mutation distributions of sequences at 12 time points. The horizontal axis represents the gene position, and the vertical axis represents the mutations of the mutant sequence compared with the reference sequence (the sequence at the first time point). For example, the scale “1” means the sequence at the first time point, and the scale “2” means the sequence at the second time point; the scale “12” means the sequence at the 12th time point; (**c**) Single nucleotide mutation frequencies of sequences at 12 time points. The horizontal axis represents the gene position, and the vertical axis represents the number of sequences mutated at the corresponding gene site. The sequence at the first time point is regarded as the reference sequence, and the mutation profiles of all genes are assembled without considering the noncoding region.

To better visualize the mutation distributions and frequencies of virus sequences over time in the same patients, we extend the Mutation Tracker method illustrated in [35,36] to present them. For the data of each patient, multiple gene sequences are aligned by MUSCLE [37,38], and a sequence at the first time point is regarded as the reference sequence. The aligned gene sequences are re-positioned and compared according to reference sequence. Gaps caused by insertion and deletion are also taken into account. Then, the mutation diversity of gene sequences is explored. Here, we assemble the mutation profiles of all genes without considering the noncoding region (the locations of the nine genes in the genomic sequence are presented in Figure 2a) and show the results in Figure 2b,c and Figures S11a,b–S19a,b. From the density of the dots in the figures, the changes in nucleotide distribution of the *Env* gene are the largest (the yellow dots are the densest when observed along the horizontal axis of Figure 2b and Figures S11a) and the mutation frequencies are the highest (the yellow bars corresponding to high mutation frequency are higher than others when observed along the longitudinal axis of Figure 2c and Figures S11b–S19b) compared with the reference sequence. This further validates the reliability of the conclusions obtained by NDDT.

### 3.2. NDDT Study of Eight Sequence Segment Regions

**Figure 3:**
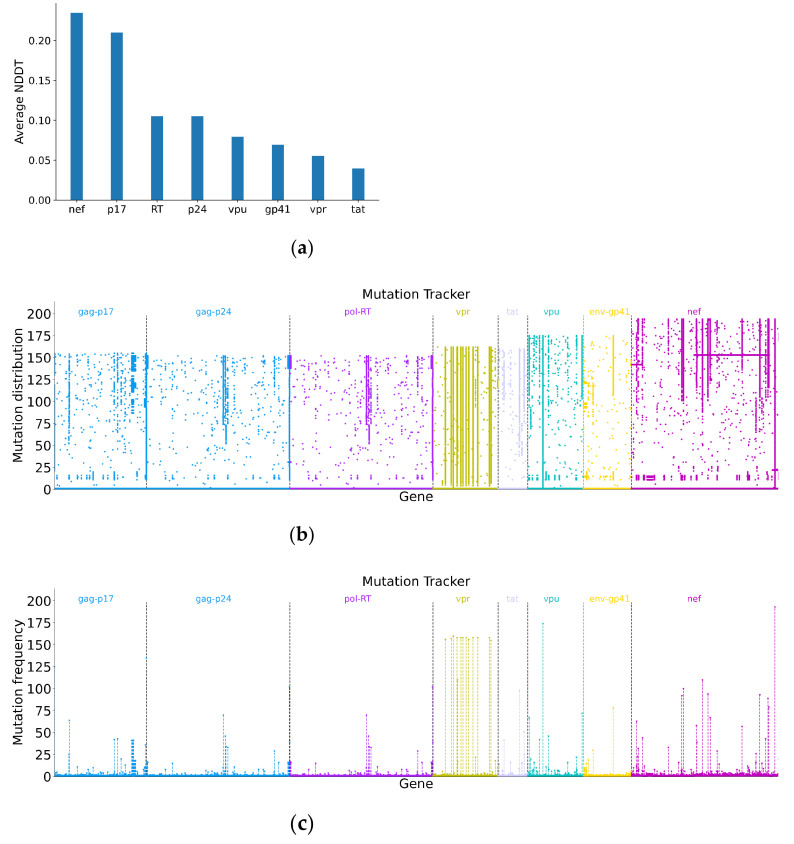
(**a**) The average nucleotide distribution difference of the eight sequence segments for patients in dataset 2 over all time periods; (**b**) Single nucleotide mutation distributions of the sequence segments at all time points; (**c**) Single nucleotide mutation frequencies of the sequence segments at all time points. The sequence at the first time point is regarded as the reference sequence.

Furthermore, we select eight sequence segment regions (*Nef*, p17, p24, RT, *Vpu*, gp41, *Vpr*, *Tat*) from the nine major genes to study the mutations. The data contain 1337 sequences provided by dataset 2. In Figure 1, *Gag*, *Pol*, *Nef* and *Vpu* show a higher average mutation rate, and *Vpr* and *Tat* show a lower average mutation rate. This result is also reflected in Figure 3a (in which segments p17 and p24 belong to *Gag* and segment RT belongs to *Pol*). The mutation rate at each time point is shown in Figure S20. Figure 3b,c further verify it by using mutation distribution and frequency. According to the sampling time points of the dataset, the nucleotide distribution changes of *Gag*, *Pol*, *Nef*, *Vpu*, *Vpr* and *Tat* in the first three years of infection have a similar pattern as those in the whole infection period.

In addition, we extract the eight sequence segments of all sequences in dataset 1, calculate the average NDDT of the eight sequence segments for each patient during the whole sampling time period, and analyze the distribution and frequency of the mutation, as shown in Figures S21–S30. In the whole infection stage, gp41 sequences of most patients show the highest mutation rate; *Nef*, *Vpu*, RT, p17 and p24 also show relatively higher average mutation rate, while *Vpr* and *Tat* show lower average mutation rate.

### 3.3. The Temporal Relationships among Nucleotide Distribution Changes, Viral Divergence, and Diversity

*Env* is an HIV gene, whose encoded protein forms the viral envelope. The *Env* gene codes for the gp160 protein, and is cut into glycoproteins gp120 and gp41. gp120 binds to the CD4 receptor on the target cell, and make the virus infiltrate the cell to bind to the co-receptor CXCR4 or CXCR5. gp41 provides the second step to let the virus enter the host cell through the target cell membrane. The first targets of HIV vaccine research are gp120 and gp41. It is vital to have a comprehensive insight into the mutation pattern of *Env* [6].

The binding of the virus and the CD4 receptor is the most obvious step during the HIV infection process. CD4 is a glycoprotein, “+” indicates positive, and CD4+ T cells are an essential part of the human immune system. If CD4 cells are depleted, the body is vulnerable to infection. It makes the relationship among virus mutation, CD4+ count and viral load become an important research direction. We explore this relationship using dataset 3.

**Figure 4:**
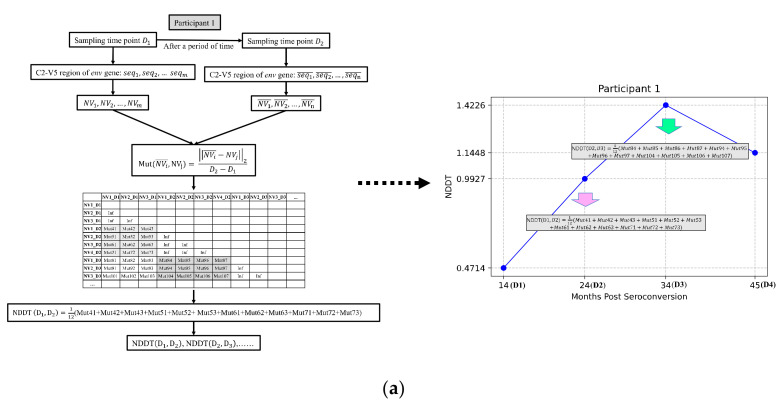

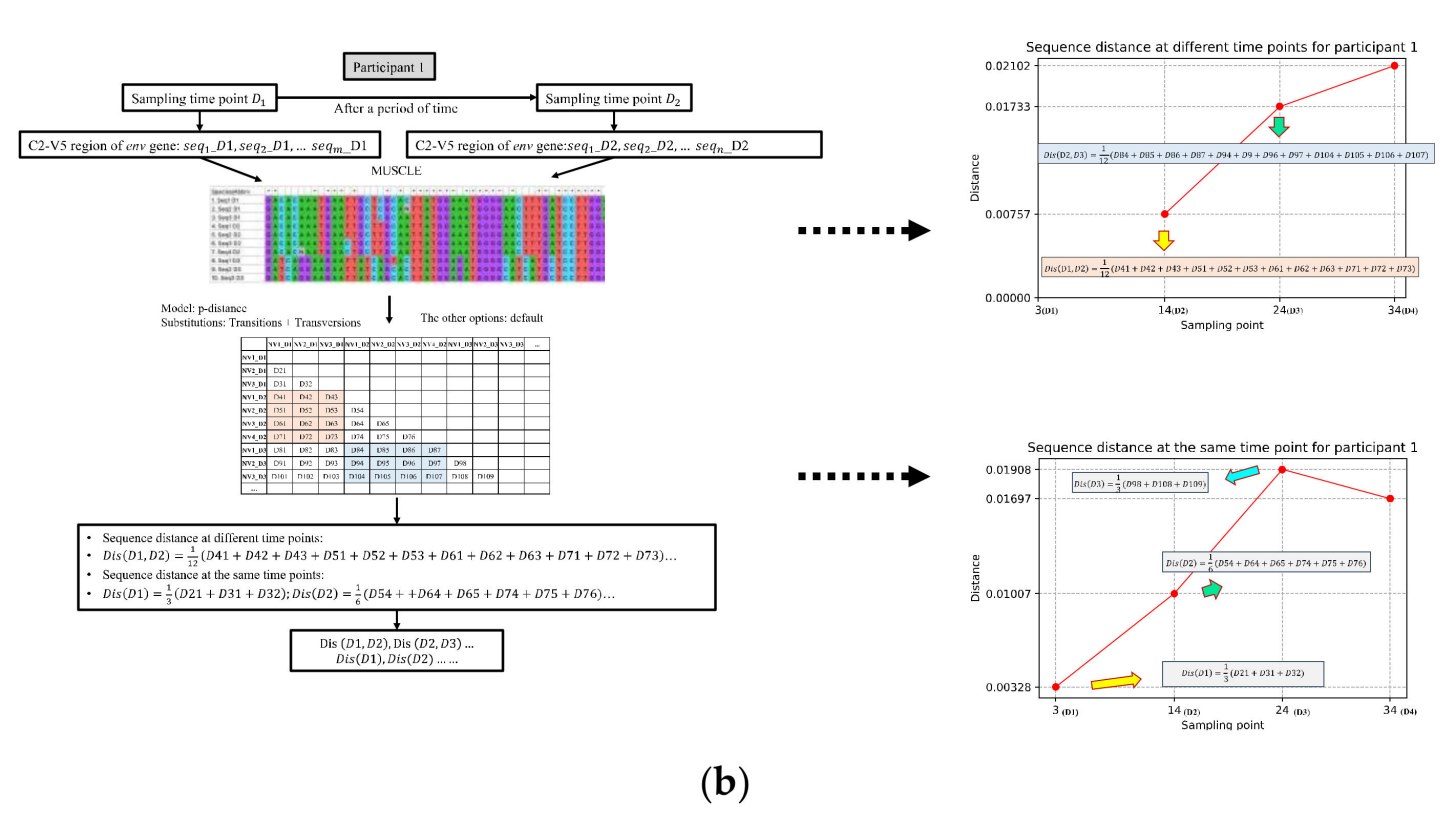
(**a**) Flowchart for computing the mean NDDT between two adjacent time points $D_{1}$ and $D_{2}$; (**b**) Flowchart for computing the viral divergence and diversity between two adjacent time points $D_{1}$ and $D_{2}$.

For participant 1, there are fifteen sampling time points—3, 14, 24, 34, 45, 51, 61, 66, 68, 77, 80, 87, 94, 98, and 105—whose unit is a month. There is more than one nucleotide sequence at each time point, and the length of the sequences at the same time point may be different. We first convert each sequence into a natural vector and then calculate NDDT. In this way, we obtain a mutation matrix and only consider the mutation sub-matrices of adjacent time points. Next, we take the average of the sub-matrix and obtain a mean mutation rate between time point $D_{1}$ and time point $D_{2}$. For each participant, we calculate the mean mutation rate between time points $D_{1}$ and $D_{2}$, and plot it at $D_{2}$ (Figure 4a).

**Figure 5:**
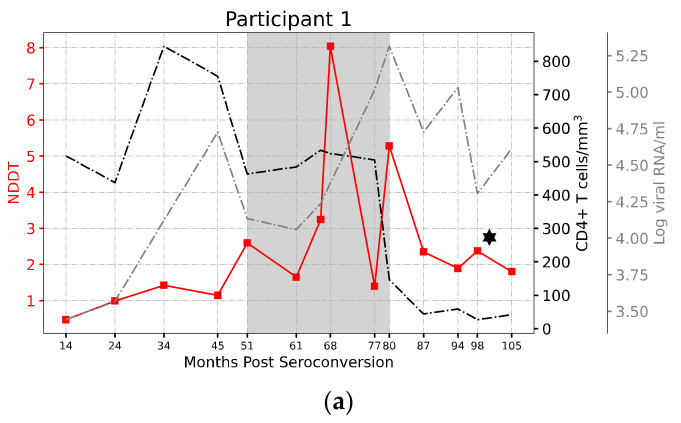

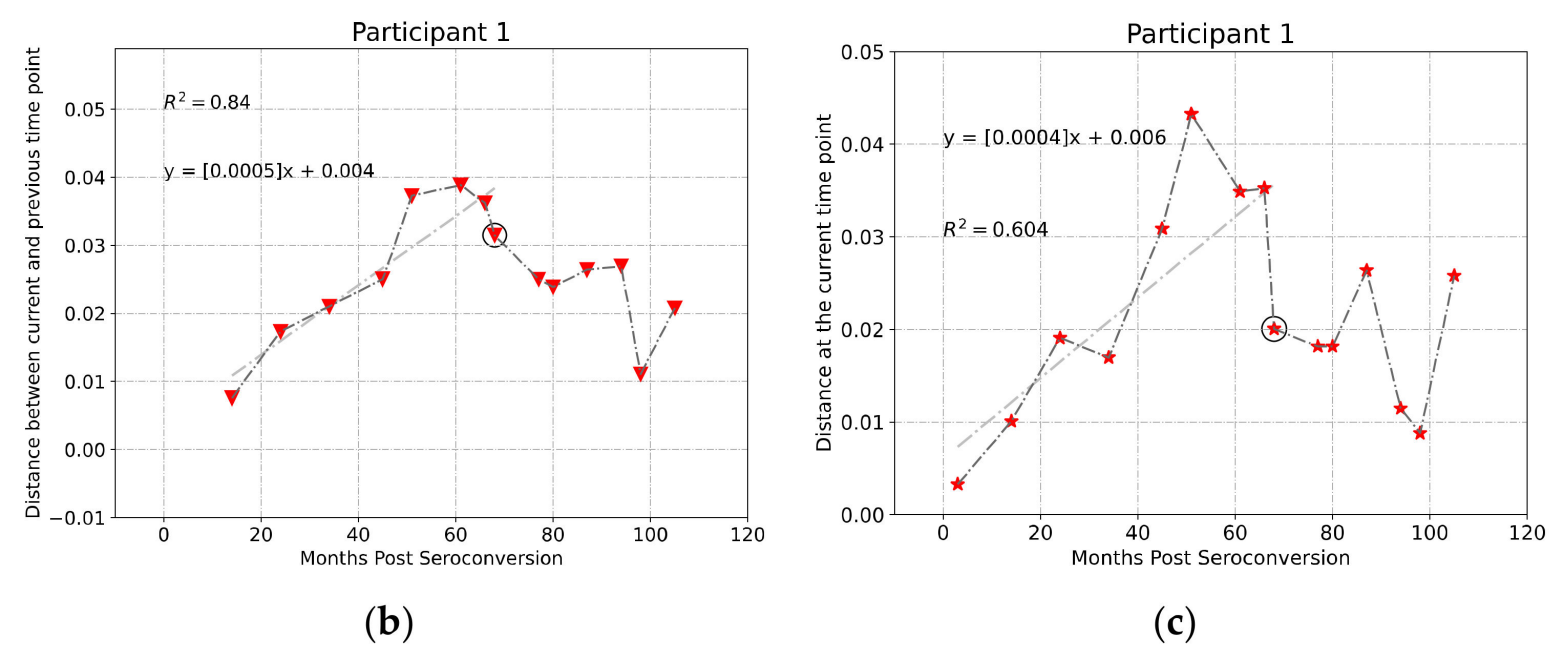
(**a**) The variation trends of C2-V5 region of HIV-1 *Env* gene for participant 1. The mutation progressions are shown with the filled blocks connected by the colorful line. The CD4+ T cell levels are shown with the black dotted line. The viral RNA levels are shown with the gray dotted line. Five participants (1, 3, 5, 6, 7) died after the last time point of analysis (marked with diamond), and their CD4+ T cell levels dropped to 200 cells/μL; (**b**) Viral divergence: Distance of sequences between the current time point and its previous time point; (**c**) Viral diversity: Distance of sequences at the current time point. The abscissa of the circle represents the time point of the mutation peak.

Figure 5a and Figures S31a–S38a show the variation trends of the C2-V5 region of the *Env* gene for the nine participants. Participants 4 and 10 are excluded for lack of specimens. The horizontal axis represents the time post seroconversion, and the three vertical axes represent the mean NDDT, CD4 count, and viral load, respectively. This provides a pattern in which viruses change over time. From the mutation trend plots (except participants 9 and 11), the NDDTs increase for several years post seroconversion, then reach a peak or several peaks, and appear to gradually slow down or decrease in the late stage of infection. The average time to the best peak is 58 months post seroconversion for all participants. Before reaching the best peak, the NDDT for each individual increases linearly (mean $R^{2}=0.55$, and mean slope=0.07, except participants 9 and 11 because their disease progressions were slow and their diseases were special cases); after the best peak, it declines linearly (mean $R^{2}=0.705$, and mean slope=−0.9, except participants 9 and 11). These facts suggest that the gene sequences relationship shows a progressive evolutionary trend, and the variation in the sequences is time-ordered, which is strong at the initial stage of infection, and then becomes weaker with the development of AIDS.

The above results are consistent with the changes in T-cell levels and viral counts. Participants 1 and 2 had high CD4+ T cell levels at the infection stage, and their NDDTs change very slowly. Participants 9 and 11 were the slowest disease progressors. The CD4 count of participant 9 was still over 200 cells/μL at the last study time point whose antiretroviral therapy began when the CD4 measure was 202 cells/μL. In the meanwhile, the mutation rate of participant 9 goes down during the initial 63 months (~5.5 years) post seroconversion, and upward after that until the last time point, which suggests that this participant was going through the disease stage. Participant 11 never received any antiretroviral therapy during all study time points, and had the highest protracted CD4 count decline. The cell count and viral load were stable for about seven years, and then were inevitably followed by a decline in CD4 and a rise in viral phenotypes in 70 months after seroconversion, which indicates that participant 11 entered the stage of disease progression.

Since it is widely accepted that results based on Multiple Sequence Alignment (MSA) are reliable, we calculate the genetic distance based on MSA at different time points or the same time point, and check whether the NDDT and the genetic distance are statistically indistinguishable, which would further support the interpretation that the NDDT and the mutation rate are strongly linked. Here, the mean genetic distance is used. The calculation flowchart in Figure 4b is described by taking participant 1 as an example, who has three sequences at time point D1, four sequences at time point D2, and three sequences at time point D3. MUSCLE is used to carry out multiple sequence alignment and the pairwise nucleotide distances among these 10 sequences are calculated. We choose the model as “p-distance”, the substitutions as “Transitions + Transversions”, and the other options by default. The above steps are implemented by MEGAX [39]. Then, a genetic distance matrix among these 10 sequences is obtained. Similarly, we compute the average distance of sequences at the current time point or the adjacent time points. Then, we can obtain insight into viral divergence and viral diversity. The viral divergence at a given time point is defined by the distance between the current time point and its previous time point, and the viral diversity at the given time point is estimated by the average genetic distance for pairwise sequences at the current time point.

Figure 5b and Figures S31b–S38b show the genetic distance changes of the virus sequences at different time points among all participants (viral divergence). The mean distance increases by about 0.36% per month for all participants when all longitudinal data are used (mean $R^{2}=0.583$). In fact, the distance increases highly linearly for the month until the NDDT reaches the highest peak (mean $R^{2}=0.876$, mean slope=0.0007, except participants 9 and 11 who are slow disease progressors) and then decreases or is stabilized later in the late stage of infection and has a less linear correlation (mean $R^{2}=0.428$, except participants 9 and 11). Figure 5c and Figures S31c–S38c show the genetic distance changes of the virus sequences at the same time point (viral diversity). The linear relationships of the mean distance for the men alive at the last study time point (mean $R^{2}=0.687$ of participants 2, 8, 9, and 11; participants 1, 3, 5, 6, and 7 died at the last study time point) are stronger than that for all men (mean $R^{2}=0.563$, mean slope=0.0333%). Furthermore, the diversity shows a strong linear correlation before the NDDT reaches the best peak (mean $R^{2}=0.784$, mean slope=0.0006, except participants 9 and 11) and a higher variability (mean $R^{2}=0.341$, except participant 9 and 11) afterwards.

To further verify the consistent pattern of virus mutation rate, viral divergence, and viral diversity, the *Env* genes of patients 1 and 3 in dataset 1 are selected for the same analysis. Patients 4 and 7 are excluded because they were suspected to superinfect and failed to amplify virus samples according to the original paper [21]. Other patients’ data are too few to be analyzed. The variation trend and the sequence distance are shown in Figure S39. For patients 1 and 3, the sequence distance between the current time points and the previous time points increases linearly ($R^{2}=0.509$ of patient 1, $R^{2}=0.781$ of patient 3) before the mutation rate reaches the peak and then decreases. The sequence distance between the current time point and the initial time point also increases linearly ($R^{2}=0.967$ of patient 1, $R^{2}=0.999$ of patient 3). It is worth noting that we redefine viral divergence, which can reflect not only the divergence trend, but also the divergence speed.

The analyses in the previous part have demonstrated the efficiency of our NDDT definition, so we used it to analyze the mutation rate of the complete genomes in dataset 1. Figure S40 shows that the NDDT during all time periods ranges from 0 to 0.2 except for patient 1. The results indicate the general applicability of our approach.

Although the time points of mutation rate change could hardly be precisely determined, we can still assert that the NDDT, CD4 count, viral load, viral divergence, and diversity have close temporal relationships and the mutation trend patterns across different individuals are highly consistent. The conclusion combining the results of sequence distances at the same or different time points provides solid evidence that our mutation rate definition is reasonable.

### 3.4. Running Time Comparison of Alignment Method and Our Alignment-Free Method

We utilize MATLAB R2019b to calculate the natural vector, and the MUSCLE algorithm of MEGAX to carry out multiple sequence alignment. The two methods are only tested on the above three datasets. For the 69 complete HIV-1 genome sequences (dataset 1), the running time of the natural vector is 1.9 s, while the time of MSA is nearly 23 min. For the 1337 sequence segment regions (dataset 2), it only takes 0.9 s to compute all natural vectors, which is faster than MSAs of all sequence segment regions. For the 1032 C2-V5 region of *Env* gene sequences (dataset 3), the total running time of natural vector is about 8.3 s, which is much less than MSA (nearly 16 min). Our alignment-free method is much faster than the MSA-based method to gain reliable results because the MSA method needs much more memory and more time to process data.

## 4. Discussion

Evolution is a function of both mutations and selective pressures (positive and negative), eliminating unfit mutations and selecting for advantageous mutations. Mutation will lead to the substitution, translocation, deletion and insertion of bases in the sequence. To describe these changes, we define the nucleotide distribution difference over time from a statistical point of view. The definition of NDDT considers not only the local properties of mutation—that is, the variations in the number and position of each base over time—but also the global properties of mutation—that is, the nucleotide distribution difference between two time points. The rationality of the definition is verified by testing on three longitudinal classical datasets. Another advantage of our method of measuring sequence differences is its great practical significance. Firstly, the sequence alignment model and genetic distance calculation model to compute the variation of mutant sequences need to be determined, but different models may lead to different results. Secondly, the sequence alignment process is very time-consuming; the aligned sequences require lots of memory to store. Our method has overcome these disadvantages, characterizing the distributions of four nucleotides of longitudinal sequences naturally and effectively, and only needing to store 12-dimensional numerical vectors. Our novel mutation representation is promising for studying mutations in a large number of longitudinal virus data.

Many details of our paper are worth discussing. Firstly, we use $l_{2}-norm$ to measure the differences of the two natural vectors. There are many other measures, but $l_{2}-norm$ has obtained satisfying results in previous sequence comparison studies [19,20,21], so this measure is intuitively applied to our study. In future work, we will consider mutations from other perspectives, for example, percent identity, mutation bias, etc. Secondly, we use three classical HIV nucleotide sequence datasets. The limited number of HIV sequences with time points might be an issue. If more reliable data of different timescales, different individuals or different groups of individuals were added into the current datasets, the results would be more persuasive. Thirdly, our conclusions in Section 3.1 and Section 3.2 are supported by prior studies in the literature, which is that the *Env* gene has the fastest evolution speed during the whole infection period [6,28]. The conclusion in Section 3.3 has been verified by the viral divergence and diversity, which have many broad applications [6,28,29,30,31], and the extended viral divergence definition can reflect not only the divergence trend, but also the divergence speed. Additionally, we found the nucleotide distribution difference over time has a close statistical relationship with viral divergence and diversity. Fourthly, we regard the sequence at the first sampling time point instead of the RefSeq (NC_001802) as the reference sequence, which can more valuably reveal the difference between the progressive sequences and the original sequence of the same individual. Fifthly, our study can be improved to compare HIV-1 subtype B to HIV-1 subtype C, or HIV-1 O group to HIV-1 M group, or perhaps other viruses with greater distances such as HCV. Sixthly, the advantage of our method for measuring nucleotide sequence differences can be applied to protein sequences; then, the natural vector is 60-dimensional because of the 20 amino acids.

The conclusion concerning mutation pattern is derived from a small number of patients; the problem of extensive applicability needs to be further explored. To start with, patients have different disease progression rates because of differences in their immune abilities. Next, other genes evolve slowly, and this mutation pattern could be different from that of *Env*. Moreover, HIV keeps evolving in infected individuals. The virus may have different variations in the host at the same time, or even mutates back to the original form, so as to survive better. Therefore, the viral variation trend might change along with time. Furthermore, the choice of data may have a great impact on the results. Some patients were superinfected or failed to amplify virus samples with low initial viral content, or received antiretroviral treatment at the study stage, which could affect the results. Some patients in the three datasets did not receive any treatment during the study period. With the advancement of medical technology, HIV medication is becoming more and more prevalent; viruses evolve rapidly and adapt to survive in their host, which makes it difficult to obtain a consistent conclusion and find a general method to study the mutation rate of HIV. Whether and how the mutation rate is affected by treatment is worthy of investigation, but requires more data and goes beyond the scope of this paper.

## 5. Conclusions

We propose a novel description based on the natural vector to measure the distributions of the mutant sequence and original sequence, and test it on three HIV datasets. The comparison of mutation rate of the nine genes is in accordance with previous research results, indicating that the *Env* gene mutates the fastest. Further analysis shows that the nucleotide distribution of several gene segment regions (*Gag*, *Pol*, *Nef*, *Vpu*) changes greatly both in the early stage of infection and in the whole infection period. The consistent patterns of the mutation rate, viral divergence and viral diversity are strongly supported by statistical analyses. The viral mutation rate can be divided into two stages during the infection period: the NDDT increases along with time in a linear manner since the infection (mean $R^{2}=0.55$), experiences high diversity and reaches a peak, and then declines or levels off at the late stage of infection (mean $R^{2}=0.705$). Before reaching the peak, the average linearly increasing time of NDDT is 4.8 years (except participants 9 and 11). The definition of NDDT is enhanced by viral divergence and diversity: The distance between the current time points and the previous time points increases highly linearly before the mutation rate reaches the peak (mean $R^{2}=0.876$); then, the virus continues to diverge for a few months and slows down or is stabilized. The genetic distances at the same time point present a strong linear correlation before the mutation rate reaches the peak (mean $R^{2}=0.784$), and then plateau or decline with more variability (mean $R^{2}=0.341$). The above results provide a basis for the rationality of the proposed NDDT and imply that the new definition could be generalized to study the progression rate of other diseases as well.

## Data Availability

The data presented in this study can be downloaded from the public database, and are also available in Supplementary Materials.

## Supplementary Materials

The following supporting information can be downloaded at: https://www.mdpi.com/article/10.3390/genes13020170/s1, Figure S1. The average nucleotide distribution difference of the nine genes for patient 1 over all time periods, Figure S2. The average nucleotide distribution difference of the nine genes for patient 2 over all time periods, Figure S3. The average nucleotide distribution difference of the nine genes for patient 3 over all time periods, Figure S4. The average nucleotide distribution difference of the nine genes for patient 4 over all time periods, Figure S5. The average nucleotide distribution difference of the nine genes for patient 5 over all time periods, Figure S6. The average nucleotide distribution difference of the nine genes for patient 6 over all time periods, Figure S7. The average nucleotide distribution difference of the nine genes for patient 7 over all time periods, Figure S8. The average nucleotide distribution difference of the nine genes for patient 8 over all time periods, Figure S9. The average nucleotide distribution difference of the nine genes for patient 9 over all time periods, Figure S10. The average nucleotide distribution difference of the nine genes for patient 11 over all time periods, Figure S11. Single nucleotide mutation distributions and frequencies of sequences at 5 time points for patient 2, Figure S12. Single nucleotide mutation distributions and frequencies of sequences at 5 time points for patient 3, Figure S13. Single nucleotide mutation distributions and frequencies of sequences at 5 time points for patient 4, Figure S14. Single nucleotide mutation distributions and frequencies of sequences at 5 time points for patient 5, Figure S15. Single nucleotide mutation distributions and frequencies of sequences at 5 time points for patient 6, Figure S16. Single nucleotide mutation distributions and frequencies of sequences at 5 time points for patient 7, Figure S17. Single nucleotide mutation distributions and frequencies of sequences at 5 time points for patient 8, Figure S18. Single nucleotide mutation distributions and frequencies of sequences at 5 time points for patient 9, Figure S19. Single nucleotide mutation distributions and frequencies of sequences at 5 time points for patient 11, Figure S20. The nucleotide distribution difference of the eight segments for patient in Dataset 2 at each time periods, Figure S21. (a) The average nucleotide distribution difference of the eight sequence segments for patient 1 in Dataset 1 over all time periods. (b) Single nucleotide mutation distributions of the sequence segments at all time points. (c) Single nucleotide mutation frequencies of the segments at all time points, Figure S22. (a) The average nucleotide distribution difference of the eight sequence segments for patient 2 in Dataset 1 over all time periods. (b) Single nucleotide mutation distributions of the sequence segments at all time points. (c) Single nucleotide mutation frequencies of the segments at all time points, Figure S23. (a) The average nucleotide distribution difference of the eight sequence segments for patient 3 in Dataset 1 over all time periods. (b) Single nucleotide mutation distributions of the sequence segments at all time points. (c) Single nucleotide mutation frequencies of the segments at all time points, Figure S24. (a) The average nucleotide distribution difference of the eight sequence segments for patient 4 in Dataset 1 over all time periods. (b) Single nucleotide mutation distributions of the sequence segments at all time points. (c) Single nucleotide mutation frequencies of the segments at all time points, Figure S25. (a) The average nucleotide distribution difference of the eight sequence segments for patient 5 in Dataset 1 over all time periods. (b) Single nucleotide mutation distributions of the sequence segments at all time points. (c) Single nucleotide mutation frequencies of the segments at all time points, Figure S26. (a) The average nucleotide distribution difference of the eight sequence segments for patient 6 in Dataset 1 over all time periods. (b) Single nucleotide mutation distributions of the sequence segments at all time points. (c) Single nucleotide mutation frequencies of the segments at all time points, Figure S27. (a) The average nucleotide distribution difference of the eight sequence segments for patient 7 in Dataset 1 over all time periods. (b) Single nucleotide mutation distributions of the sequence segments at all time points. (c) Single nucleotide mutation frequencies of the segments at all time points, Figure S28. (a) The average nucleotide distribution difference of the eight sequence segments for patient 8 in Dataset 1 over all time periods. (b) Single nucleotide mutation distributions of the sequence segments at all time points. (c) Single nucleotide mutation frequencies of the segments at all time points, Figure S29. (a) The average nucleotide distribution difference of the eight sequence segments for patient 9 in Dataset 1 over all time periods. (b) Single nucleotide mutation distributions of the sequence segments at all time points. (c) Single nucleotide mutation frequencies of the segments at all time points, Figure S30. (a) The average nucleotide distribution difference of the eight sequence segments for patient 11 in Dataset 1 over all time periods. (b) Single nucleotide mutation distributions of the sequence segments at all time points. (c) Single nucleotide mutation frequencies of the segments at all time points, Figure S31. (a) The variation trends of C2-V5 region of HIV-1 env gene for participant 2. The mutation progressions are shown with the filled blocks connected by the colorful line. The CD4+ T cell levels are shown with the black dotted line. The viral RNA levels are with the gray dotted line. (b) Viral divergence: Distance of sequences between the current time point and its previous time point. (c) Viral diversity: Distance of sequences at the current time point, Figure S32. (a) The variation trends of C2-V5 region of HIV-1 env gene for participant 3. The mutation progressions are shown with the filled blocks connected by the colorful line. The CD4+ T cell levels are shown with the black dotted line. The viral RNA levels are with the gray dotted line. Participant 3 died after the last time point of analysis (marked with diamond) (b) Viral divergence: Distance of sequences between the current time point and its previous time point. (c) Viral diversity: Distance of sequences at the current time point, Figure S33. (a) The variation trends of C2-V5 region of HIV-1 env gene for participant 5. The mutation progressions are shown with the filled blocks connected by the colorful line. The CD4+ T cell levels are shown with the black dotted line. The viral RNA levels are with the gray dotted line. Participant 5 died after the last time point of analysis (marked with diamond) (b) Viral divergence: Distance of sequences between the current time point and its previous time point. (c) Viral diversity: Distance of sequences at the current time point, Figure S34. (a) The variation trends of C2-V5 region of HIV-1 env gene for participant 6. The mutation progressions are shown with the filled blocks connected by the colorful line. The CD4+ T cell levels are shown with the black dotted line. The viral RNA levels are with the gray dotted line. Participant 6 died after the last time point of analysis (marked with diamond) (b) Viral divergence: Distance of sequences between the current time point and its previous time point. (c) Viral diversity: Distance of sequences at the current time point, Figure S35. (a) The variation trends of C2-V5 region of HIV-1 env gene for participant 7. The mutation progressions are shown with the filled blocks connected by the colorful line. The CD4+ T cell levels are shown with the black dotted line. The viral RNA levels are with the gray dotted line. Participant 7 died after the last time point of analysis (marked with diamond) (b) Viral divergence: Distance of sequences between the current time point and its previous time point. (c) Viral diversity: Distance of sequences at the current time point, Figure S36. (a) The variation trends of C2-V5 region of HIV-1 env gene for participant 8. The mutation progressions are shown with the filled blocks connected by the colorful line. The CD4+ T cell levels are shown with the black dotted line. The viral RNA levels are with the gray dotted line. (b) Viral divergence: Distance of sequences between the current time point and its previous time point. (c) Viral diversity: Distance of sequences at the current time point, Figure S37. (a) The variation trends of C2-V5 region of HIV-1 env gene for participant 9. The mutation progressions are shown with the filled blocks connected by the colorful line. The CD4+ T cell levels are shown with the black dotted line. The viral RNA levels are with the gray dotted line. (b) Viral divergence: Distance of sequences between the current time point and its previous time point. (c) Viral diversity: Distance of sequences at the current time point, Figure S38. (a) The variation trends of C2-V5 region of HIV-1 env gene for participant 11. The mutation progressions are shown with the filled blocks connected by the colorful line. The CD4+ T cell levels are shown with the black dotted line. The viral RNA levels are with the gray dotted line. (b) Viral divergence: Distance of sequences between the current time point and its previous time point. (c) Viral diversity: Distance of sequences at the current time point, Figure S39. (a) Mutation rate of Env gene sequence. (b) Sequence distance between the current time point and its previous time point. (c) Sequence distance between the current time point and the initial time point, Figure S40. Mutation rate analysis of complete genomes. Datasets S1 to S3.
