# Supplementary material for: Identification of HIV Rapid Mutations Using Differences in Nucleotide Distribution over Time
Source: Genes (Basel). 2022 Jan 19;13(2):170. doi: 10.3390/genes13020170 (PMC8872422; doi:10.3390/genes13020170)
Supplement: Supplementary file 1 [file genes-13-00170-s001.zip › genes-1506403-Supplementary.pdf]

Supplementary information for

Identification of HIV rapid mutations using differences in nucleotide distribution over time

Nan Sun [1], Jie Yang [2] and Stephen S.-T. Yau [1,3,*]

[1] Department of Mathematical Sciences, Tsinghua University, Beijing, China; sunn19@mails.tsinghua.edu.cn
[2] Department of Mathematics, Statistics, and Computer Science, University of Illinois at Chicago, Chicago, USA; jyang06@uic.edu
[3] Yanqi Lake Beijing Institute of Mathematical Sciences and Applications, Beijing, China; yau@uic.edu
* Correspondence: yau@uic.edu

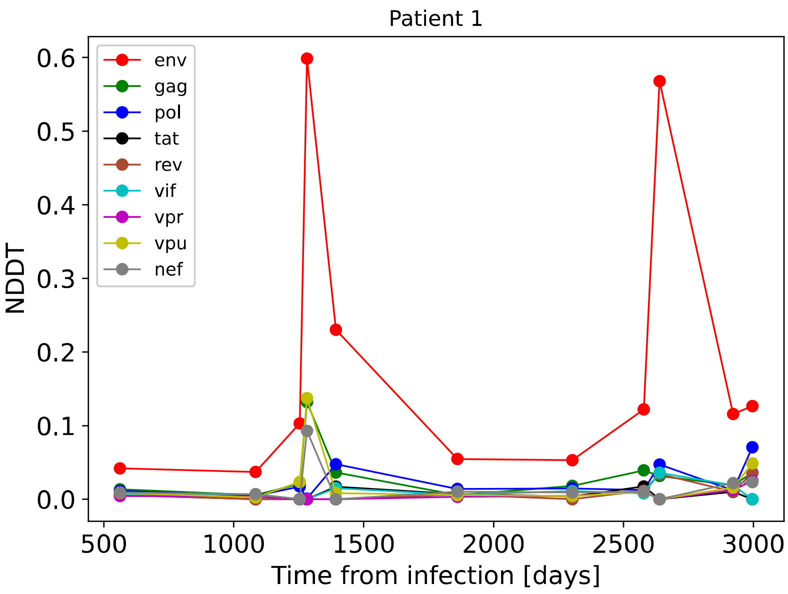

Figure S1. The average nucleotide distribution difference of the nine genes for patient 1 over all time periods.

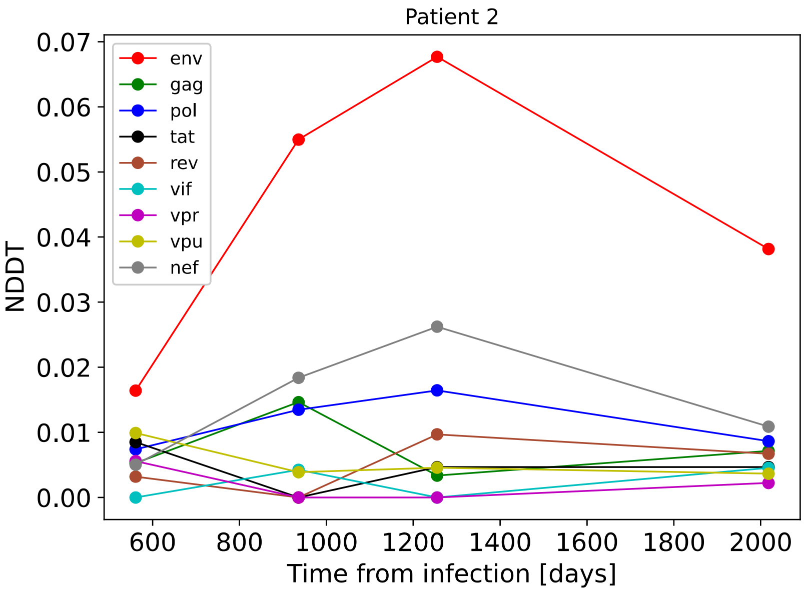

Figure S2. The average nucleotide distribution difference of the nine genes for patient 2 over all time periods.

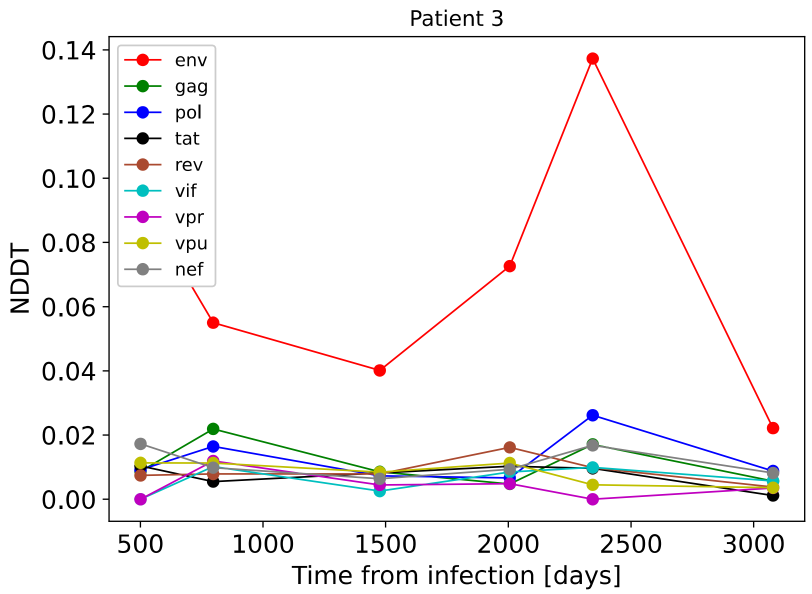

Figure S3. The average nucleotide distribution difference of the nine genes for patient 3 over all time periods.

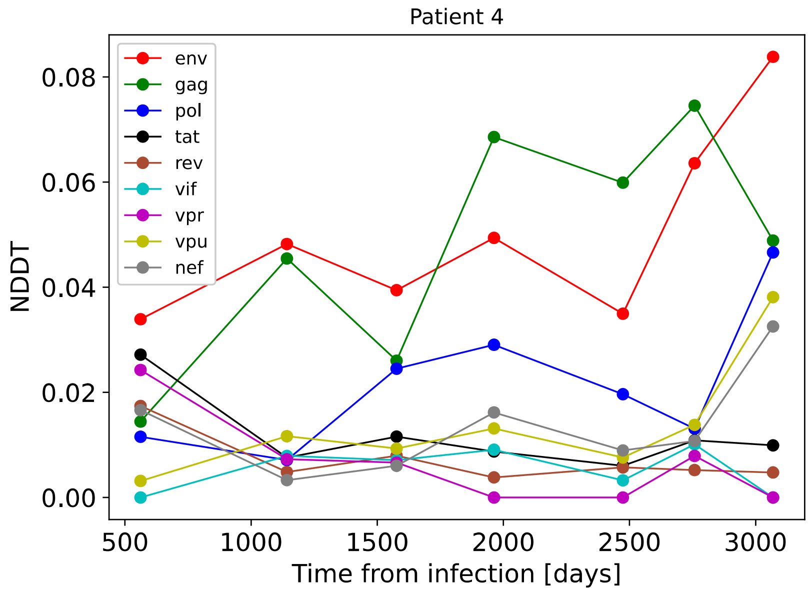

Figure S4. The average nucleotide distribution difference of the nine genes for patient 4 over all time periods.

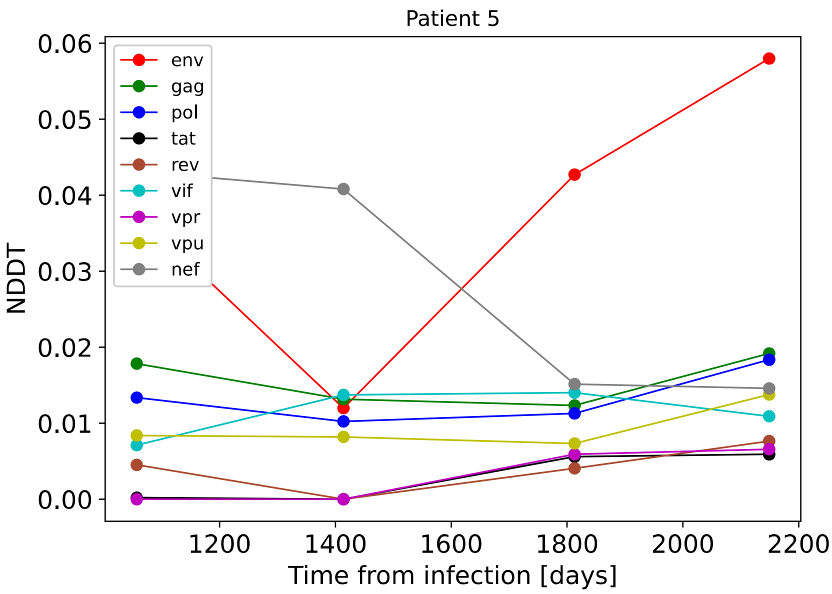

Figure S5. The average nucleotide distribution difference of the nine genes for patient 5 over all time periods.

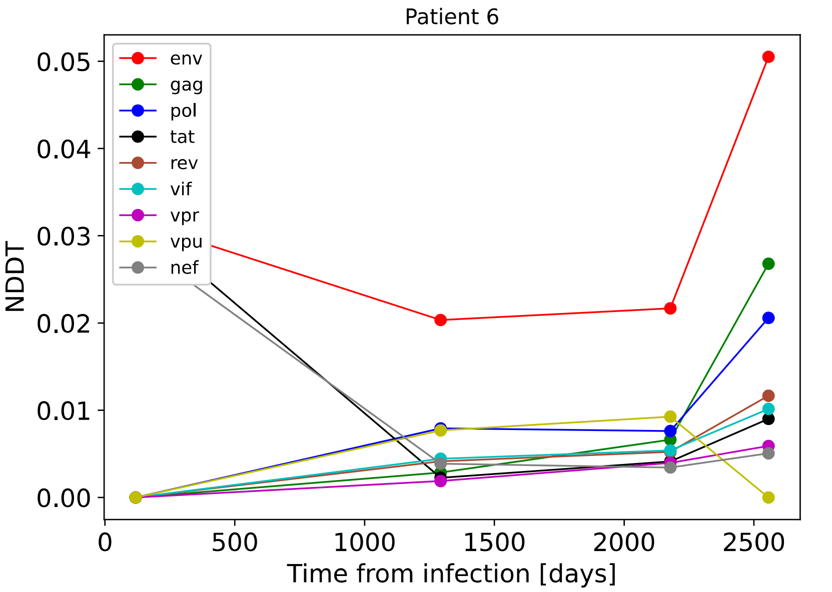

Figure S6. The average nucleotide distribution difference of the nine genes for patient 6 over all time periods.

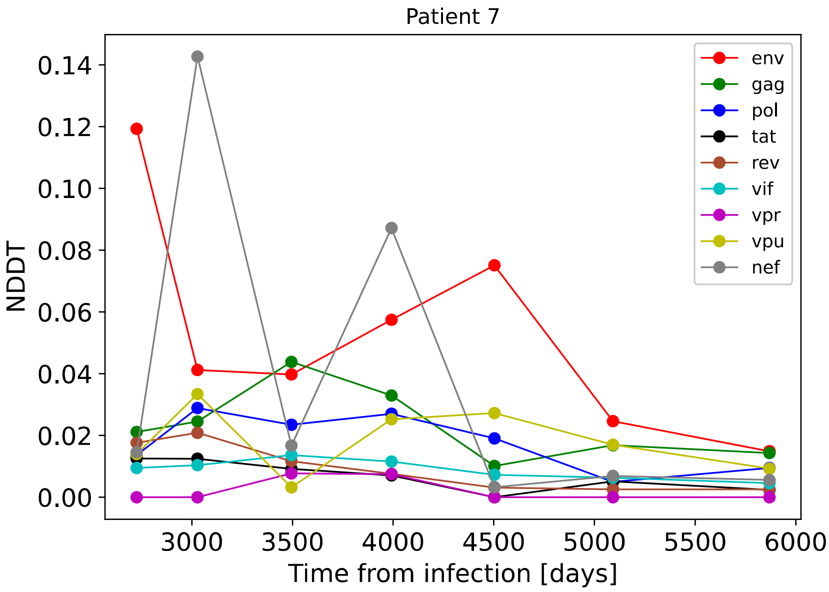

Figure S7. The average nucleotide distribution difference of the nine genes for patient 7 over all time periods.

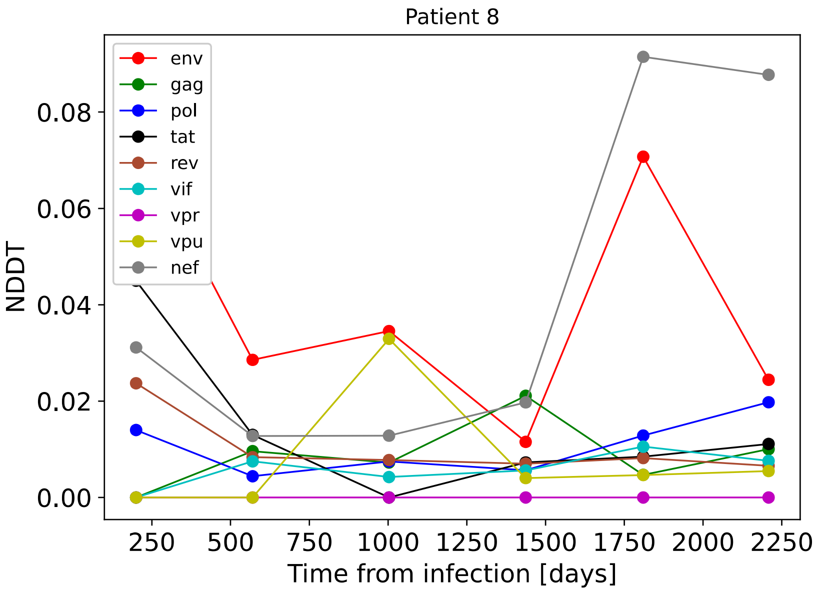

Figure S8. The average nucleotide distribution difference of the nine genes for patient 8 over all time periods.

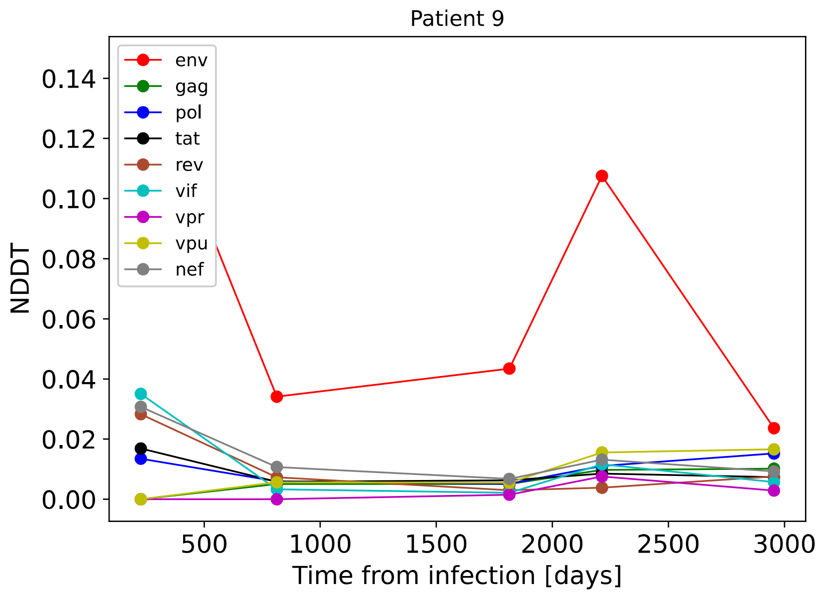

Figure S9. The average nucleotide distribution difference of the nine genes for patient 9 over all time periods.

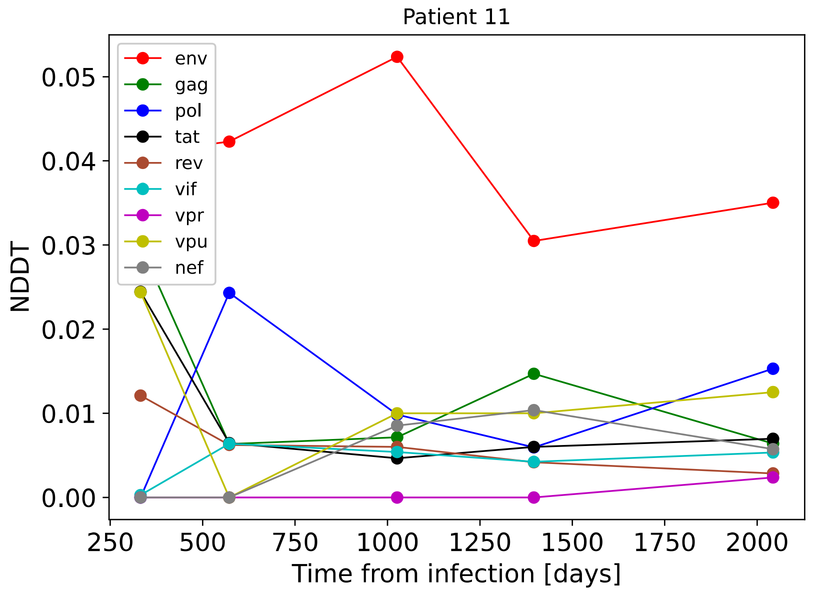

Figure S10. The average nucleotide distribution difference of the nine genes for patient 11 over all time periods.

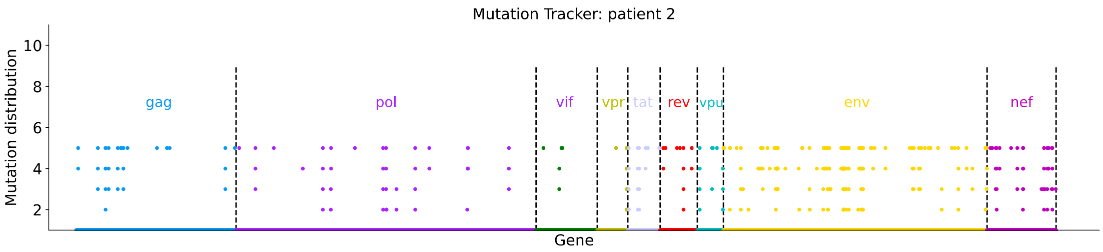

(a)

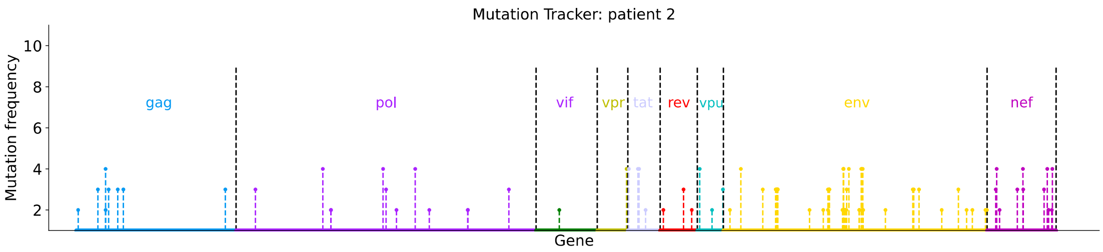

(b)

Figure S11. (a) Single nucleotide mutation distributions of sequences at 5 time points. (b) Single nucleotide mutation frequencies of sequences at 5 time points. The sequence at the first time point is regarded as reference sequence, and the mutation profiles of all genes are assembled without considering the noncoding region.

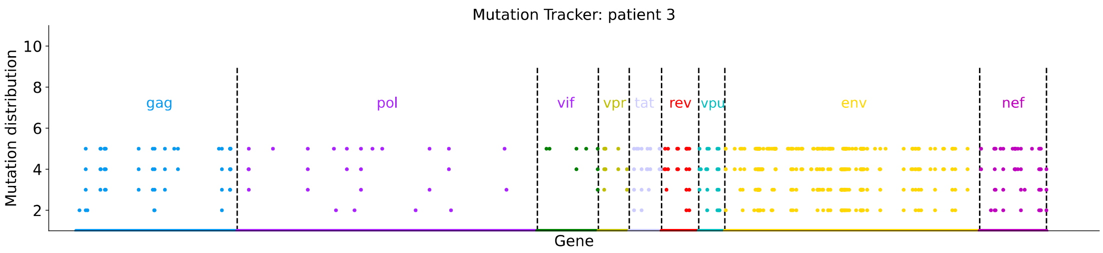

(a)

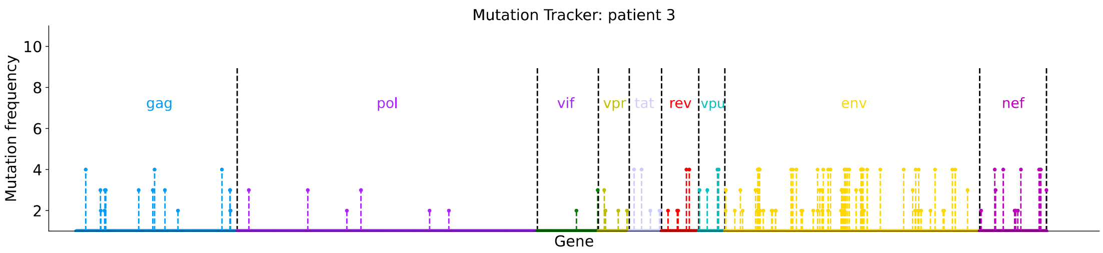

(b)

Figure S12. (a) Single nucleotide mutation distributions of sequences at 5 time points. (b) Single nucleotide mutation frequencies of sequences at 5 time points. The sequence at the first time point is regarded as reference sequence, and the mutation profiles of all genes are assembled without considering the noncoding region.

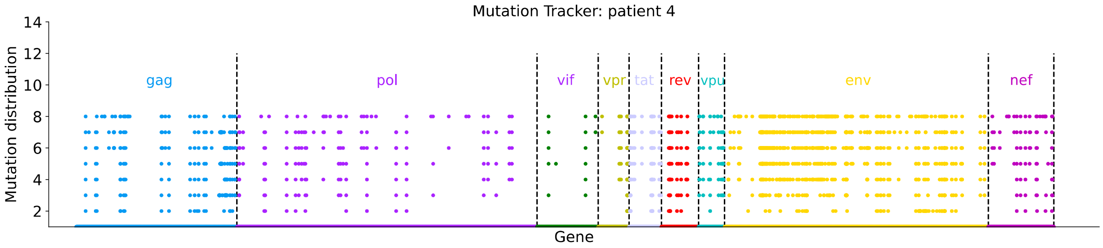

(a)

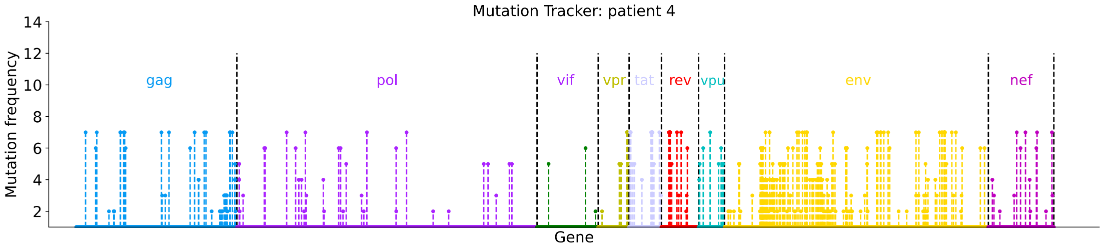

(b)

Figure S13. (a) Single nucleotide mutation distributions of sequences at 8 time points. (b) Single nucleotide mutation frequencies of sequences at 8 time points. The sequence at the first time point is regarded as reference sequence, and the mutation profiles of all genes are assembled without considering the noncoding region.

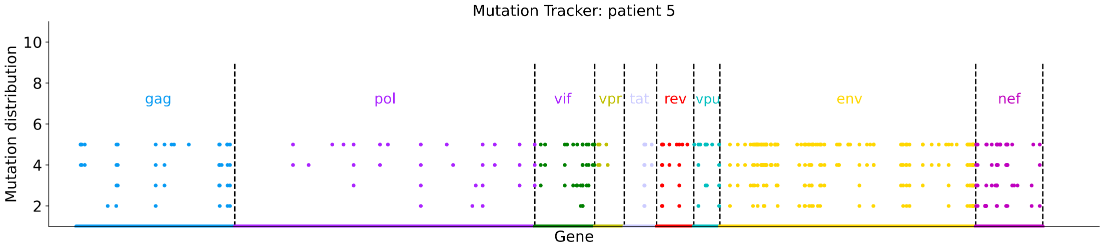

(a)

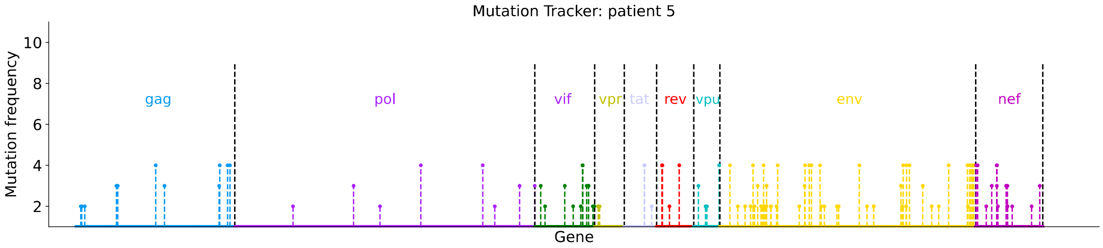

(b)

Figure S14. (a) Single nucleotide mutation distributions of sequences at 5 time points. (b) Single nucleotide mutation frequencies of sequences at 5 time points. The sequence at the first time point is regarded as reference sequence, and the mutation profiles of all genes are assembled without considering the noncoding region.

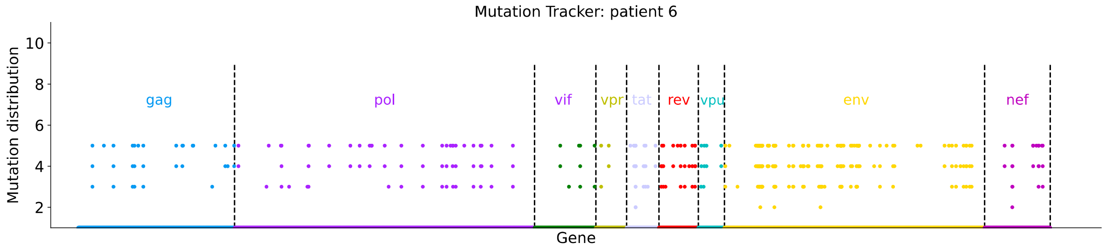

(a)

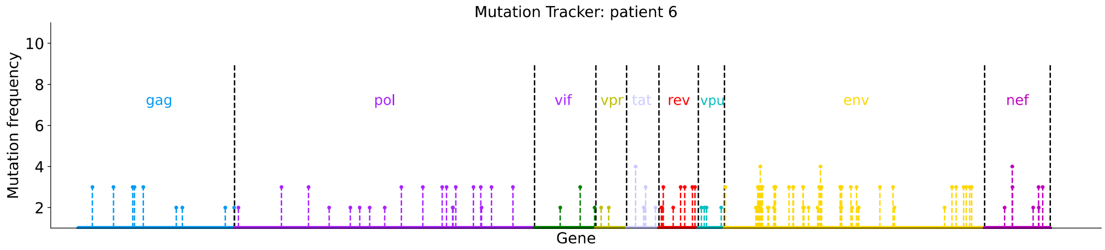

(b)

Figure S15. (a) Single nucleotide mutation distributions of sequences at 5 time points. (b) Single nucleotide mutation frequencies of sequences at 5 time points. The sequence at the first time point is regarded as reference sequence, and the mutation profiles of all genes are assembled without considering the noncoding region.

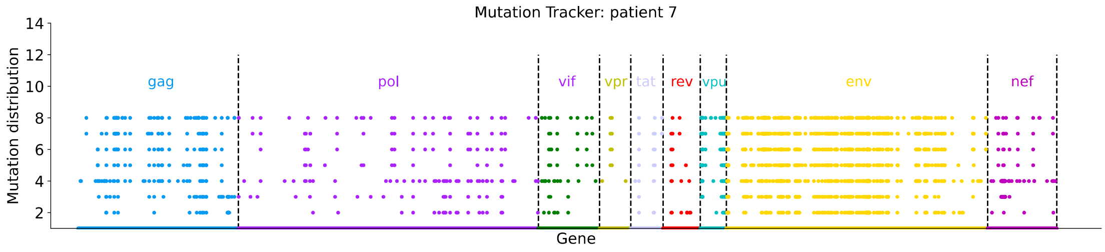

(a)

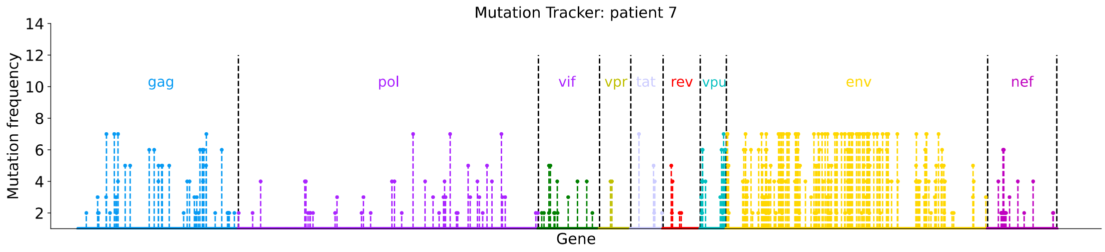

(b)

Figure S16. (a) Single nucleotide mutation distributions of sequences at 8 time points. (b) Single nucleotide mutation frequencies of sequences at 8 time points. The sequence at the first time point is regarded as reference sequence, and the mutation profiles of all genes are assembled without considering the noncoding region.

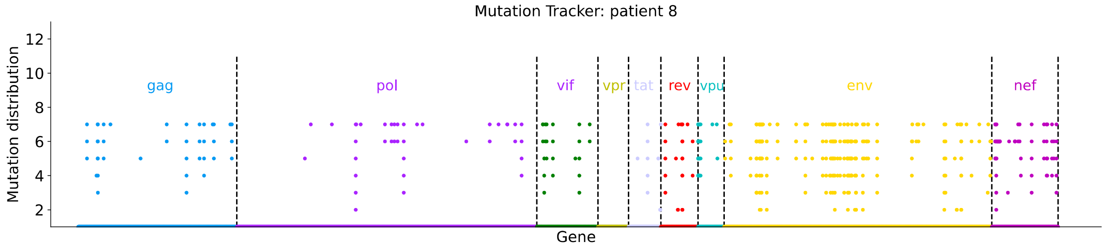

(a)

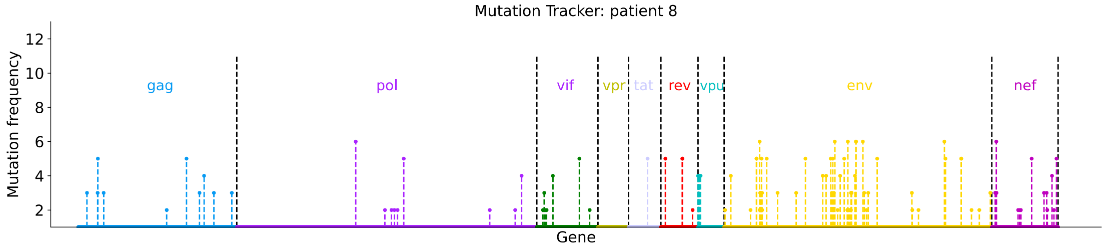

(b)

Figure S17. (a) Single nucleotide mutation distributions of sequences at 7 time points. (b) Single nucleotide mutation frequencies of sequences at 7 time points. The sequence at the first time point is regarded as reference sequence, and the mutation profiles of all genes are assembled without considering the noncoding region.

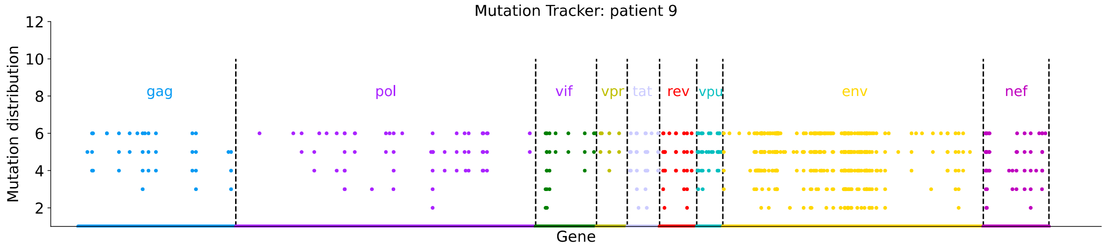

(a)

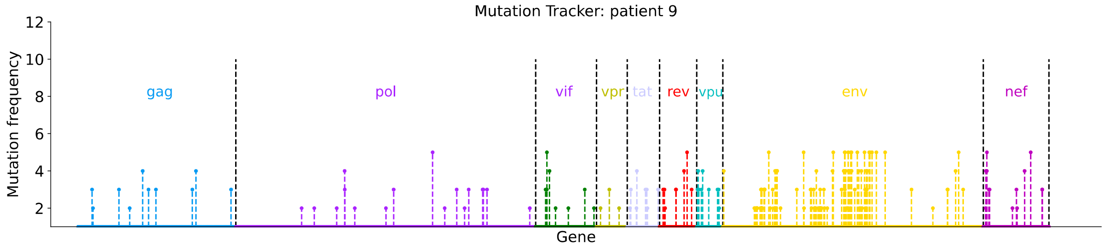

(b)

Figure S18. (a) Single nucleotide mutation distributions of sequences at 6 time points. (b) Single nucleotide mutation frequencies of sequences at 6 time points. The sequence at the first time point is regarded as reference sequence, and the mutation profiles of all genes are assembled without considering the noncoding region.

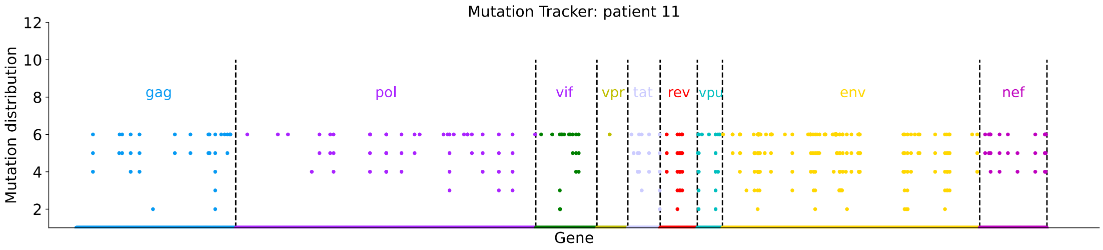

(a)

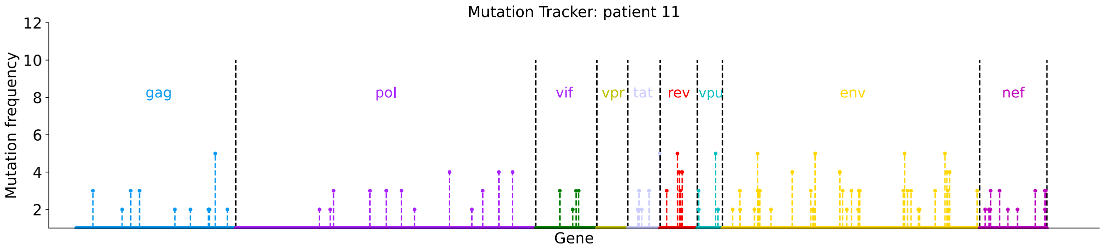

(b)

Figure S19. (a) Single nucleotide mutation distributions of sequences at 6 time points. (b) Single nucleotide mutation frequencies of sequences at 6 time points. The sequence at the first time point is regarded as reference sequence, and the mutation profiles of all genes are assembled without considering the noncoding region.

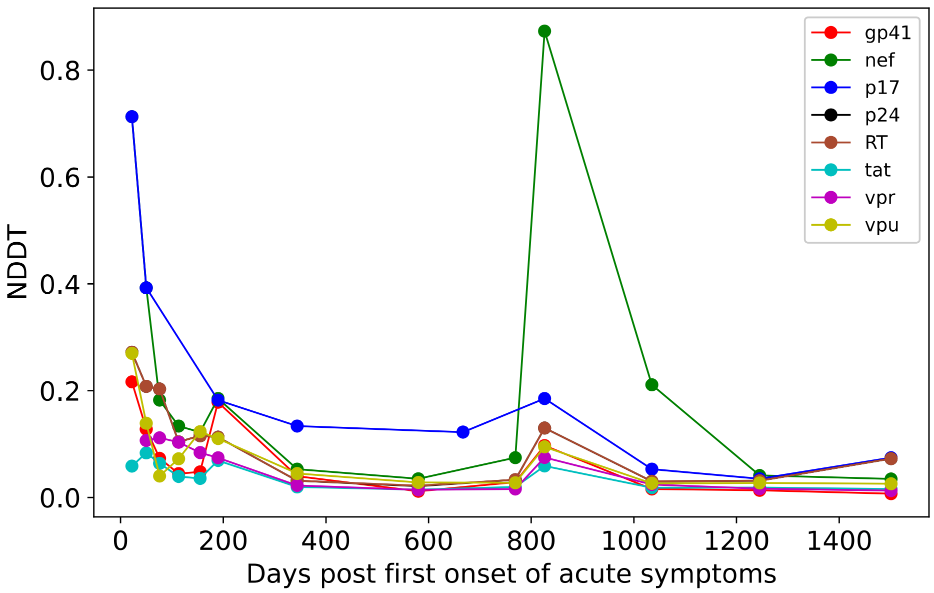

Figure S20. The nucleotide distribution difference of the eight segments for patient in Dataset 2 at each time periods.

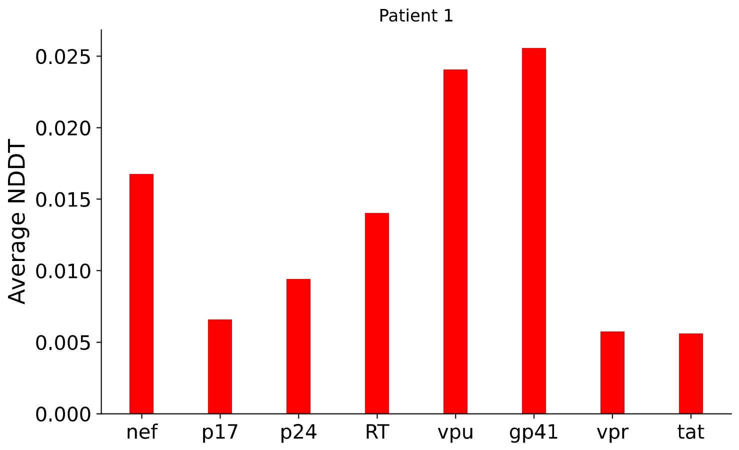

(a)

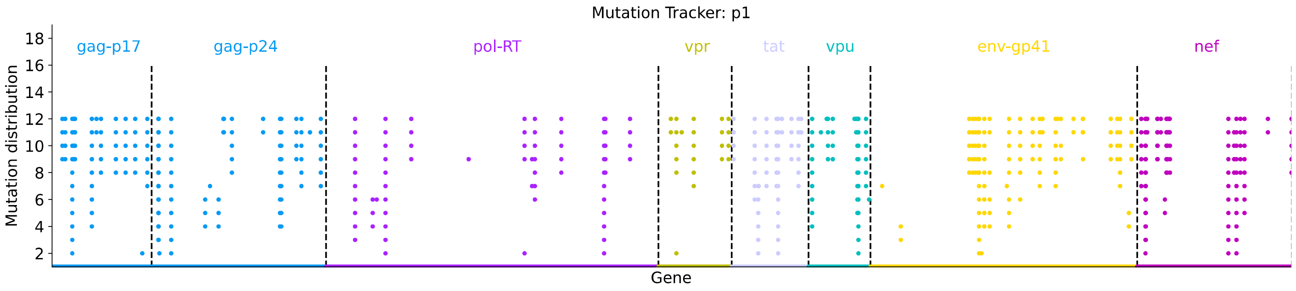

(b)

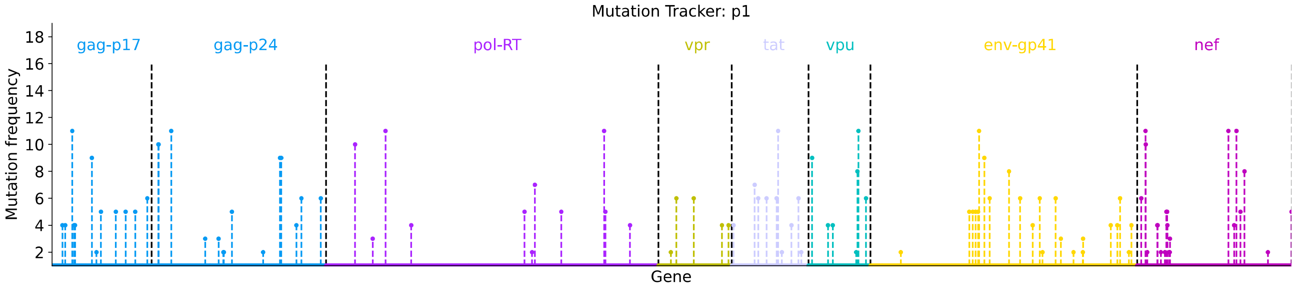

(c)

Figure S21. (a) The average nucleotide distribution difference of the eight sequence segments for patient 1 in Dataset 1 over all time periods. (b) Single nucleotide mutation distributions of the sequence segments at all time points. (c) Single nucleotide mutation frequencies of the segments at all time points. The sequence at the first time point is regarded as reference sequence.

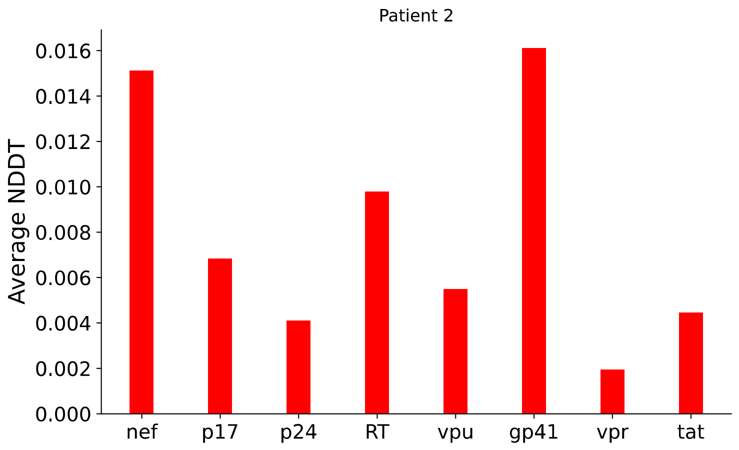

(a)

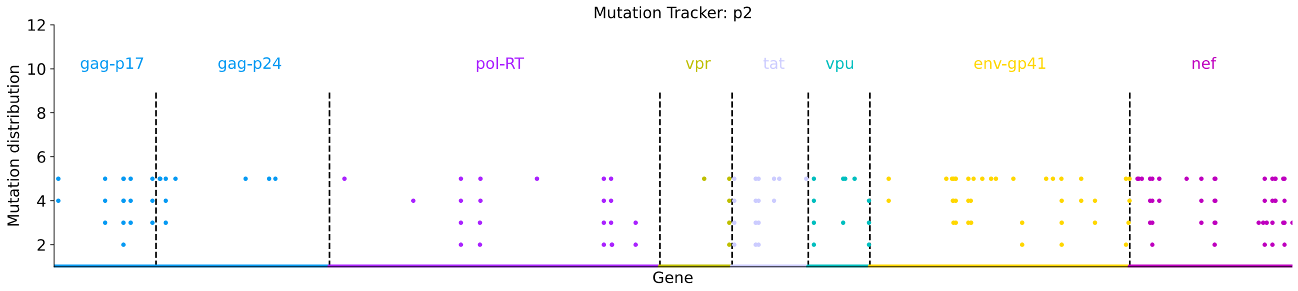

(b)

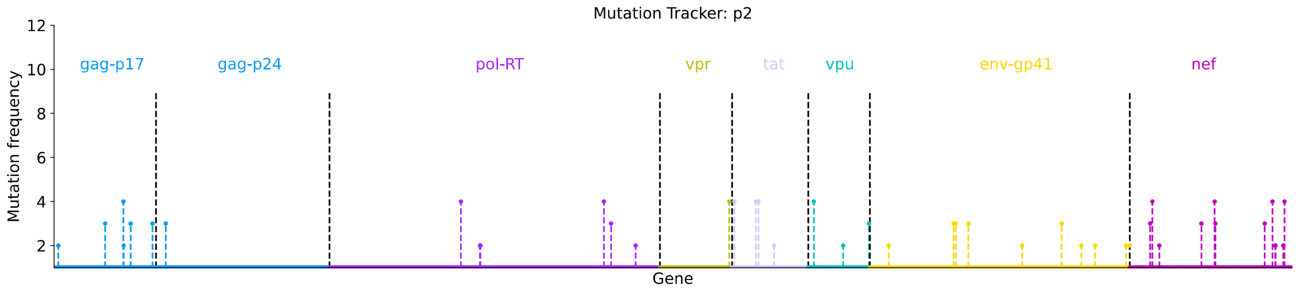

(c)

Figure S22. (a) The average nucleotide distribution difference of the eight sequence segments for patient 2 in Dataset 1 over all time periods. (b) Single nucleotide mutation distributions of the sequence segments at all time points. (c) Single nucleotide mutation frequencies of the segments at all time points. The sequence at the first time point is regarded as reference sequence.

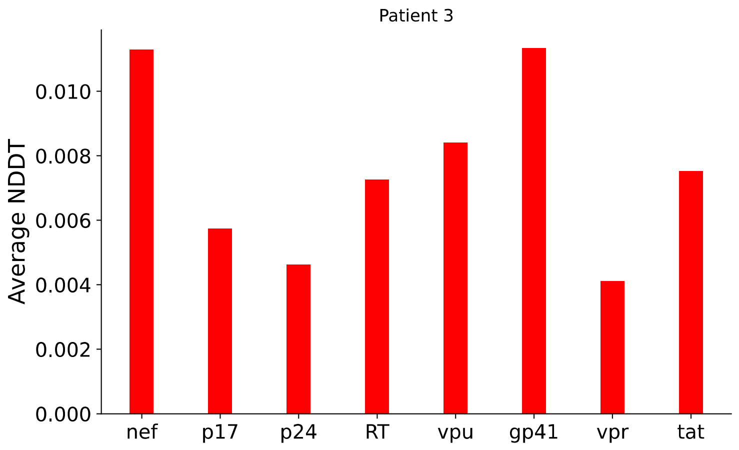

(a)

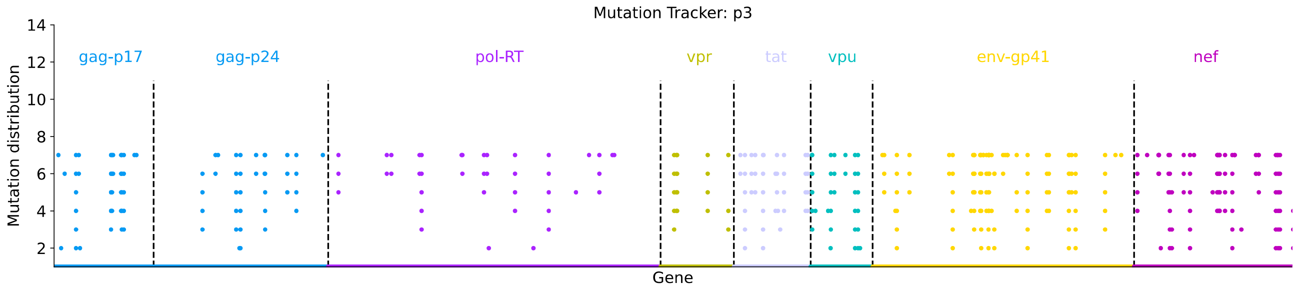

(b)

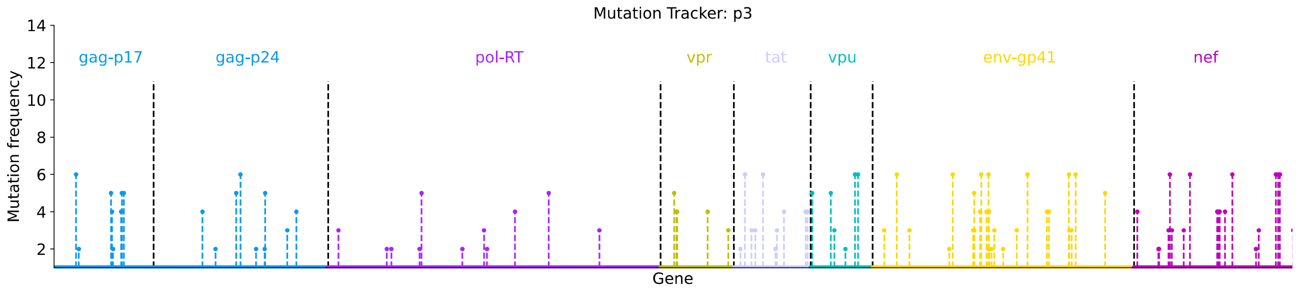

(c)

Figure S23. (a) The average nucleotide distribution difference of the eight sequence segments for patient 3 in Dataset 1 over all time periods. (b) Single nucleotide mutation distributions of the sequence segments at all time points. (c) Single nucleotide mutation frequencies of the segments at all time points. The sequence at the first time point is regarded as reference sequence.

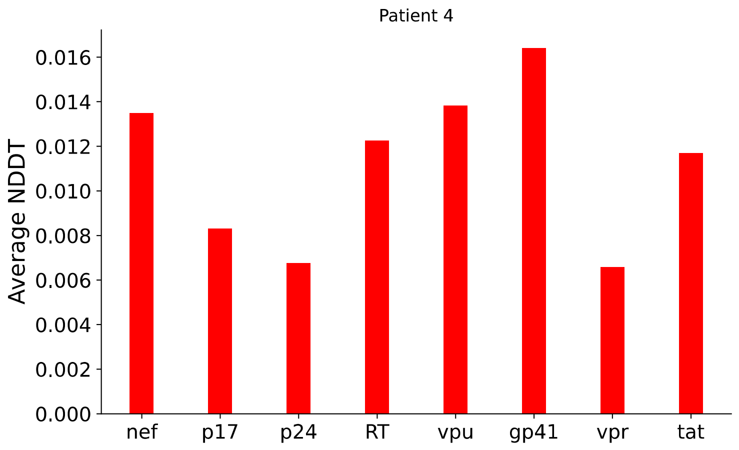

(a)

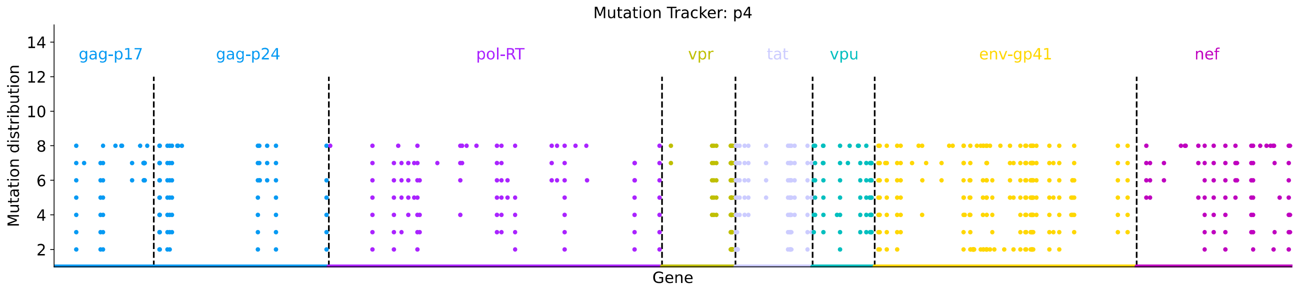

(b)

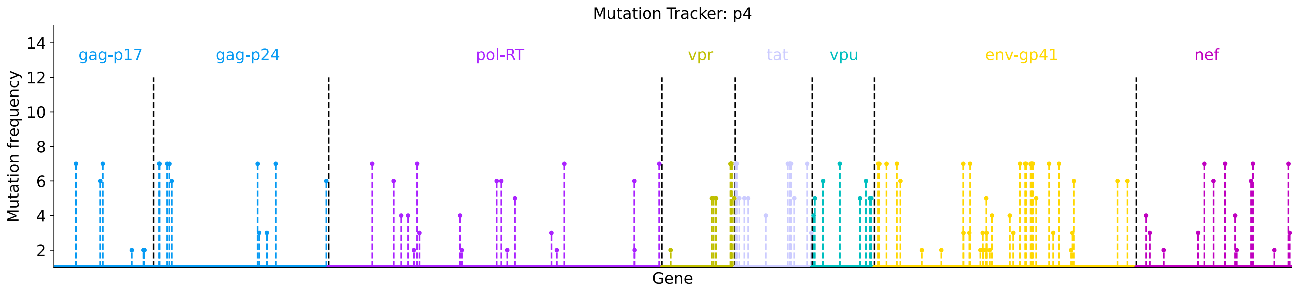

(c)

Figure S24. (a) The average nucleotide distribution difference of the eight sequence segments for patient 4 in Dataset 1 over all time periods. (b) Single nucleotide mutation distributions of the sequence segments at all time points. (c) Single nucleotide mutation frequencies of the segments at all time points. The sequence at the first time point is regarded as reference sequence.

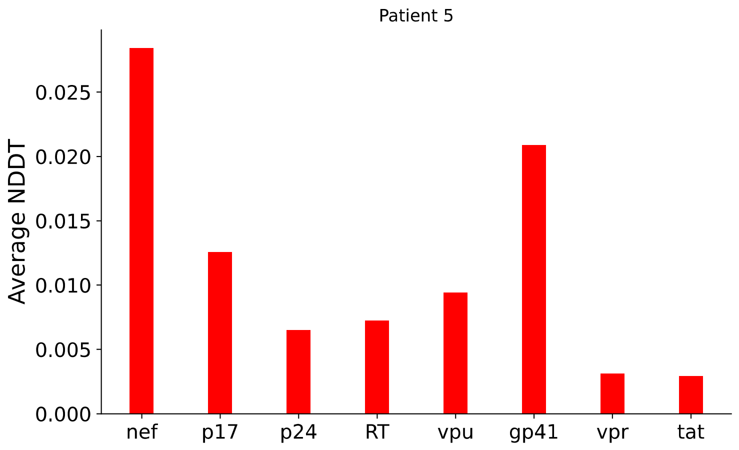

(a)

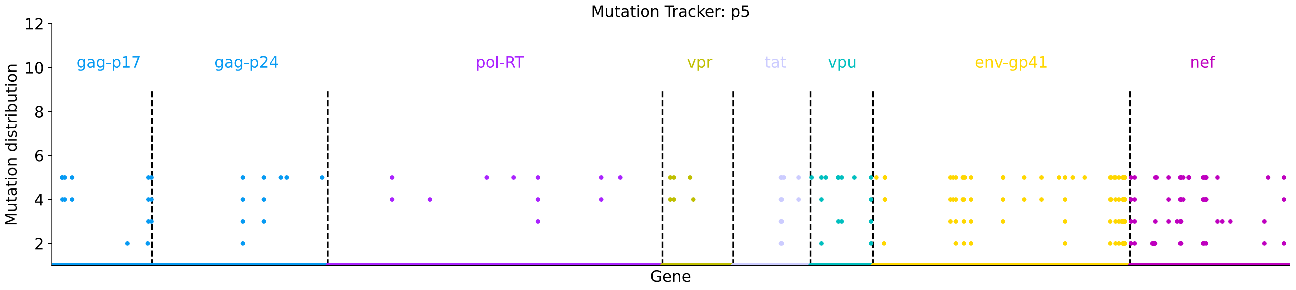

(b)

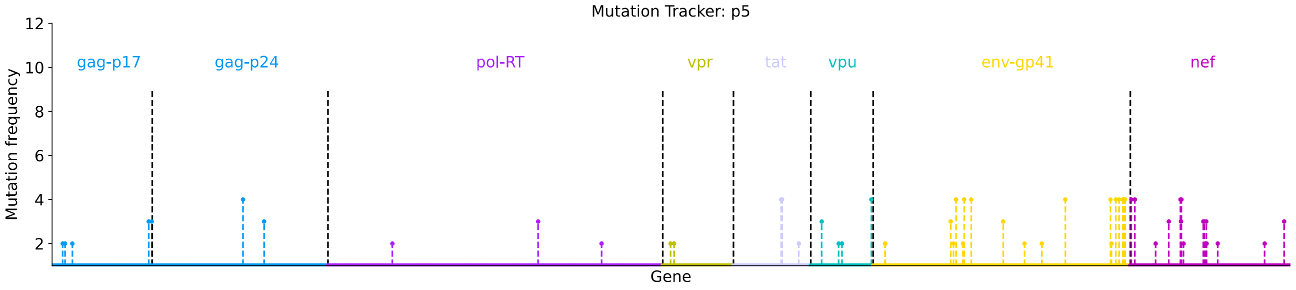

(c)

Figure S25. (a) The average nucleotide distribution difference of the eight sequence segments for patient 5 in Dataset 1 over all time periods. (b) Single nucleotide mutation distributions of the sequence segments at all time points. (c) Single nucleotide mutation frequencies of the segments at all time points. The sequence at the first time point is regarded as reference sequence.

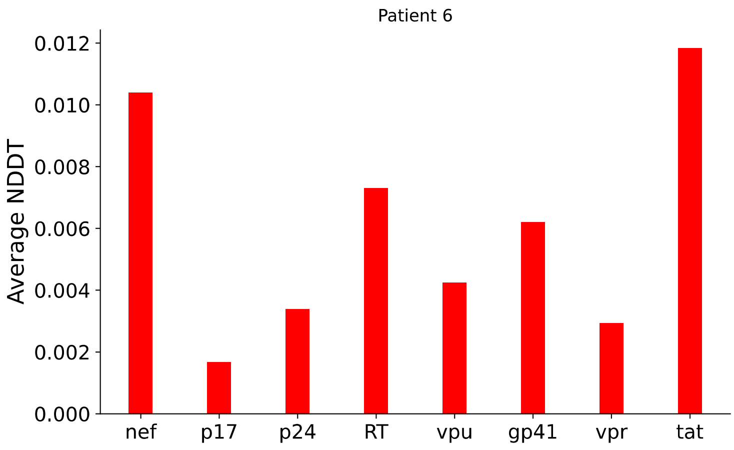

(a)

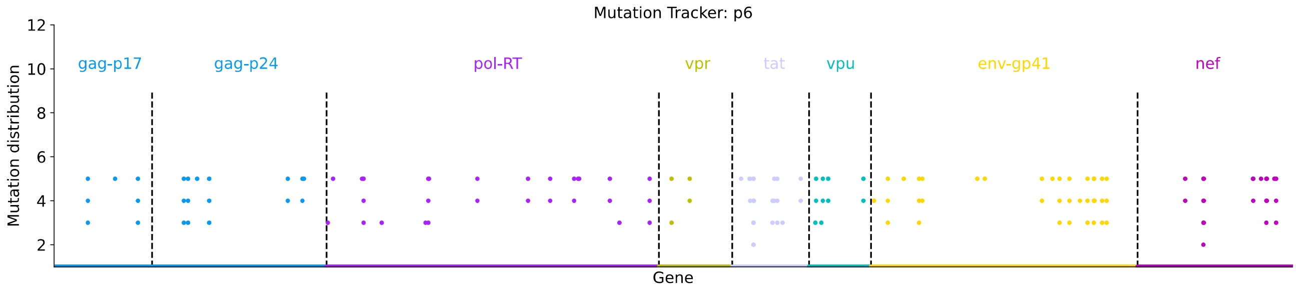

(b)

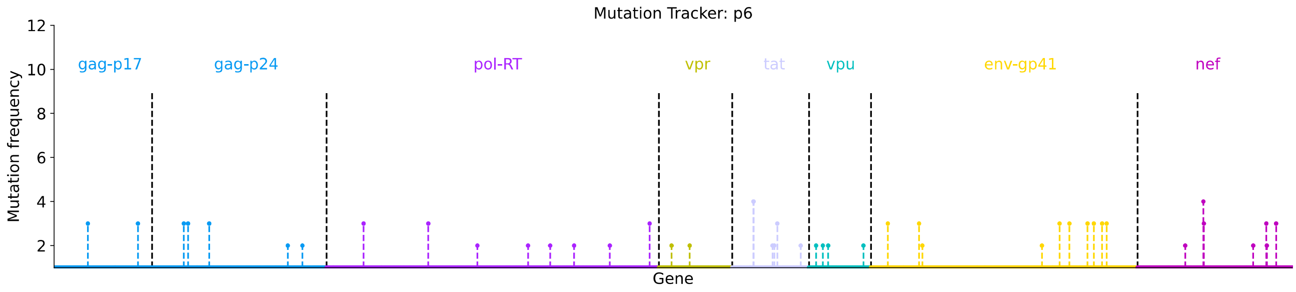

(c)

Figure S26. (a) The average nucleotide distribution difference of the eight sequence segments for patient 6 in Dataset 1 over all time periods. (b) Single nucleotide mutation distributions of the sequence segments at all time points. (c) Single nucleotide mutation frequencies of the segments at all time points. The sequence at the first time point is regarded as reference sequence.

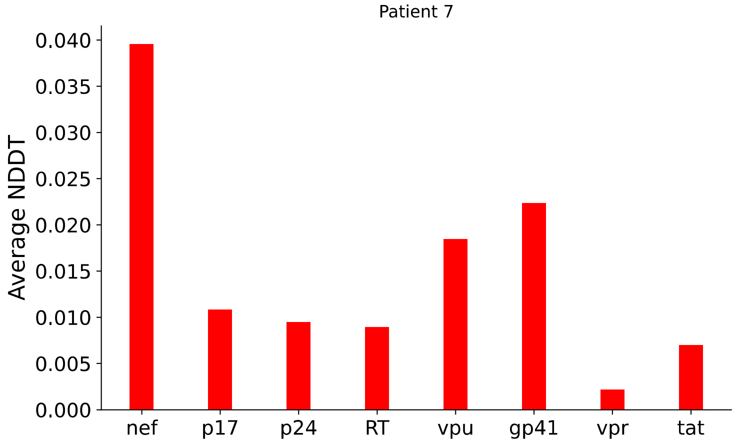

(a)

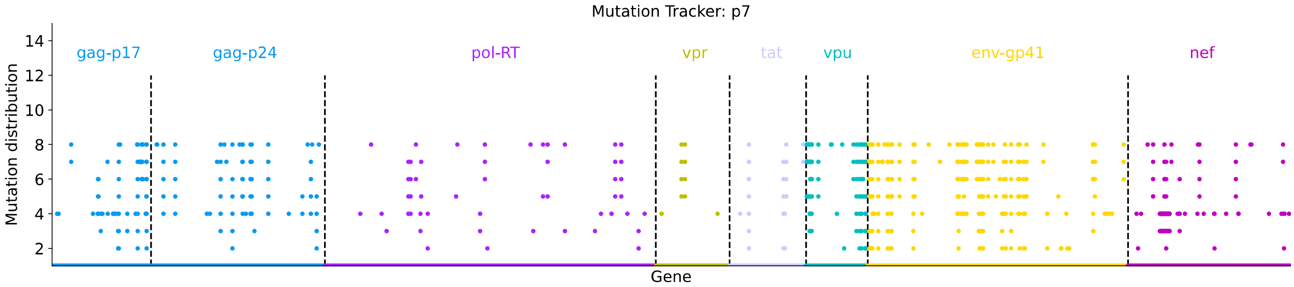

(b)

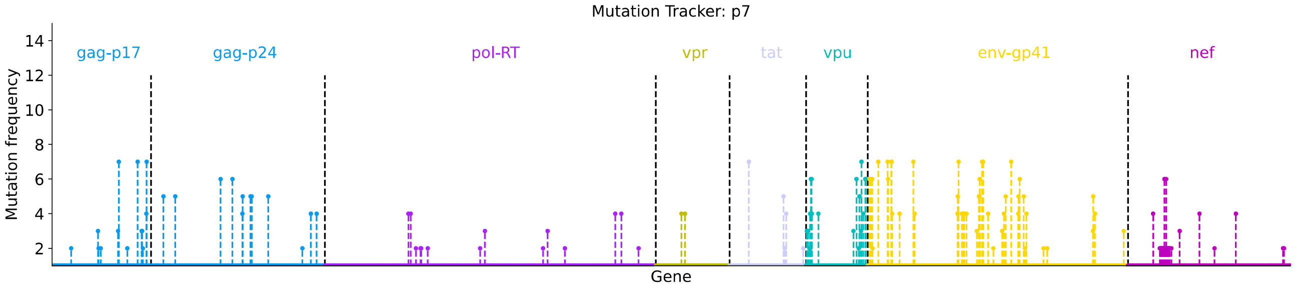

(c)

Figure S27. (a) The average nucleotide distribution difference of the eight sequence segments for patient 7 in Dataset 1 over all time periods. (b) Single nucleotide mutation distributions of the sequence segments at all time points. (c) Single nucleotide mutation frequencies of the segments at all time points. The sequence at the first time point is regarded as reference sequence.

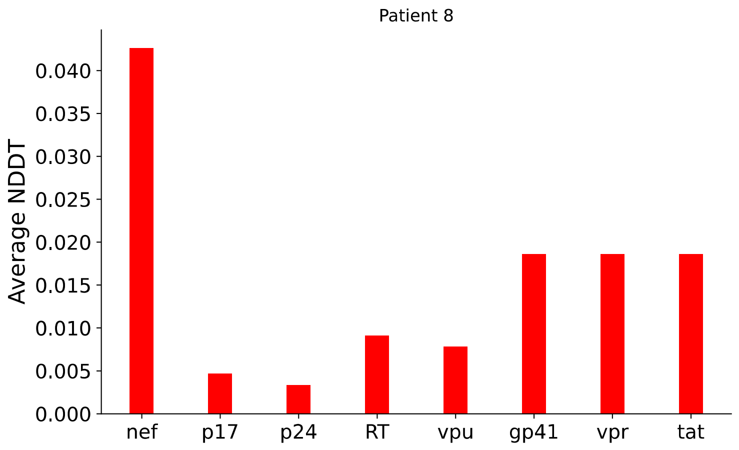

(a)

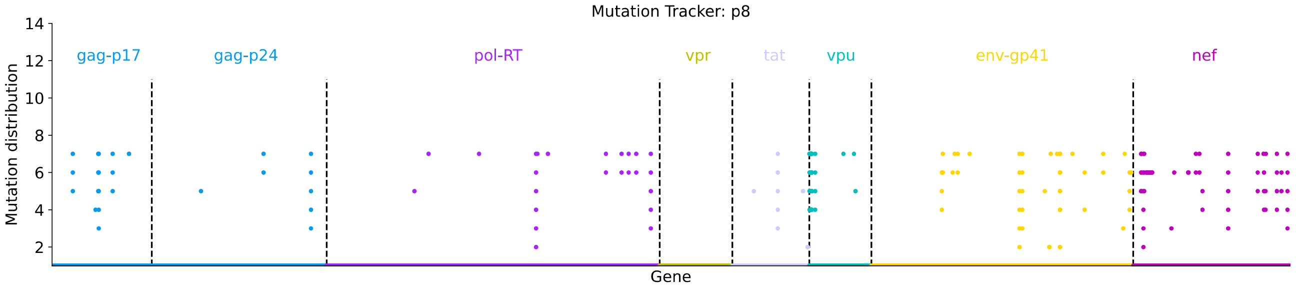

(b)

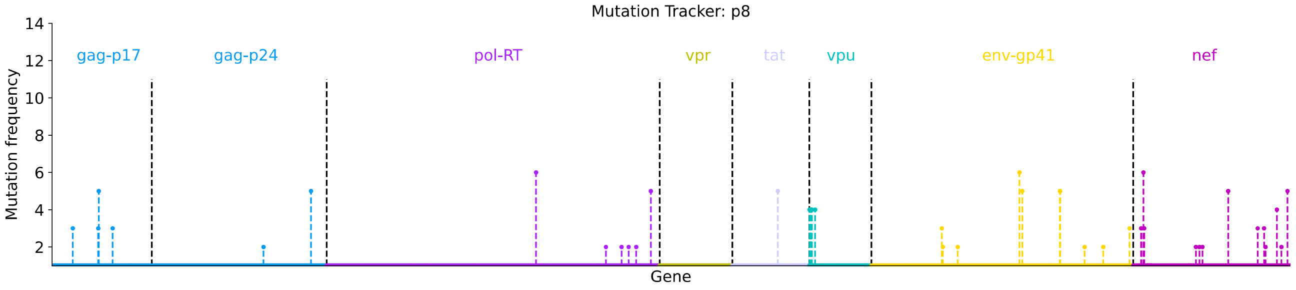

(c)

Figure S28. (a) The average nucleotide distribution difference of the eight sequence segments for patient 8 in Dataset 1 over all time periods. (b) Single nucleotide mutation distributions of the sequence segments at all time points. (c) Single nucleotide mutation frequencies of the segments at all time points. The sequence at the first time point is regarded as reference sequence.

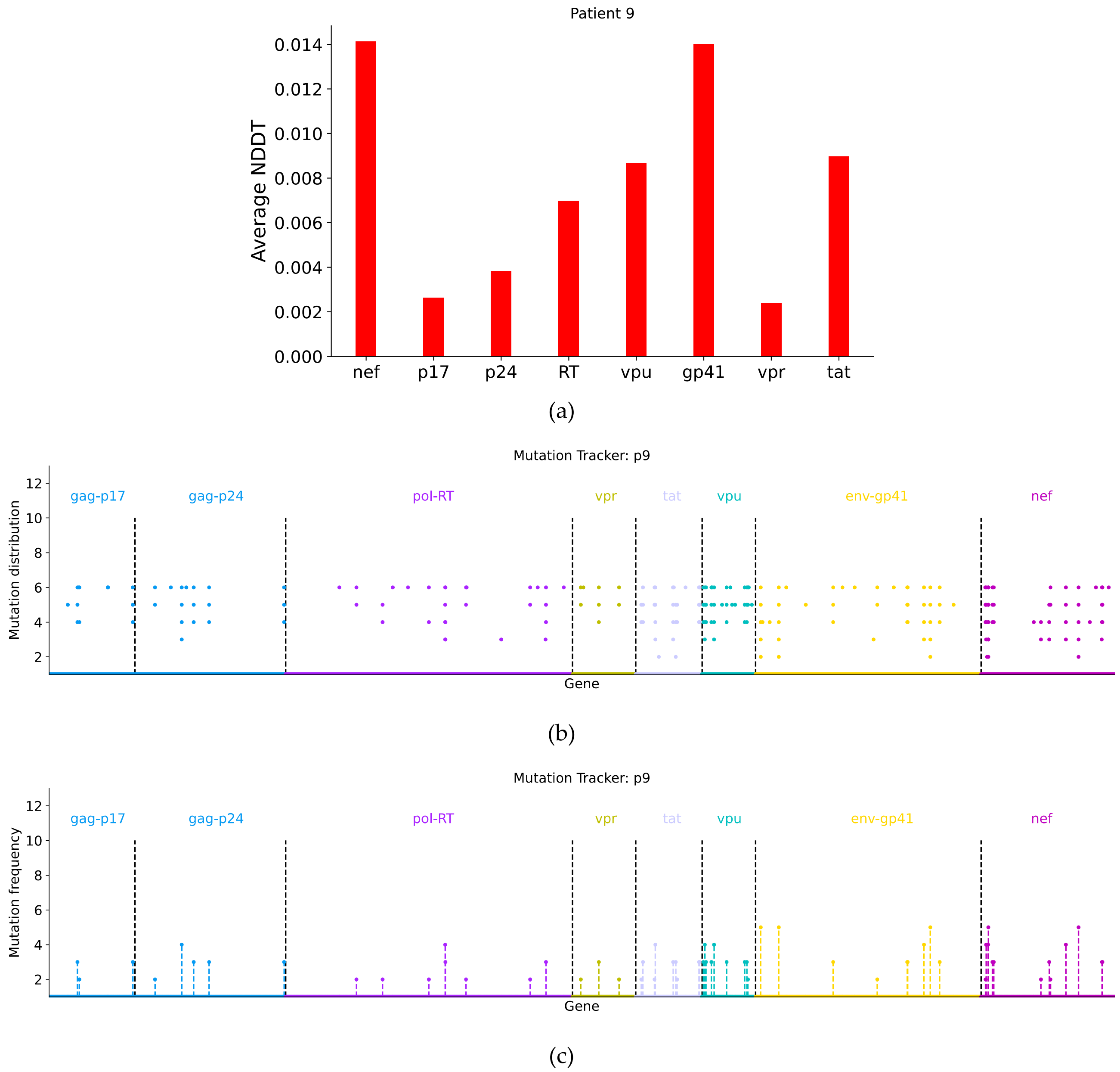

Figure S29. (a) The average nucleotide distribution difference of the eight sequence segments for patient 9 in Dataset 1 over all time periods. (b) Single nucleotide mutation distributions of the sequence segments at all time points. (c) Single nucleotide mutation frequencies of the segments at all time points. The sequence at the first time point is regarded as reference sequence.

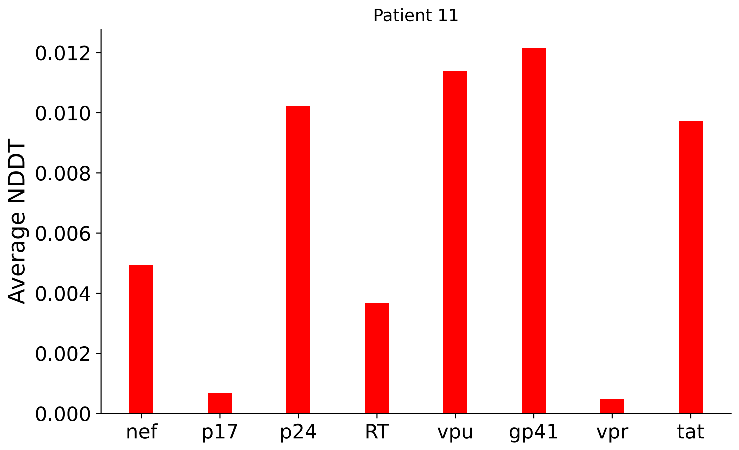

(a)

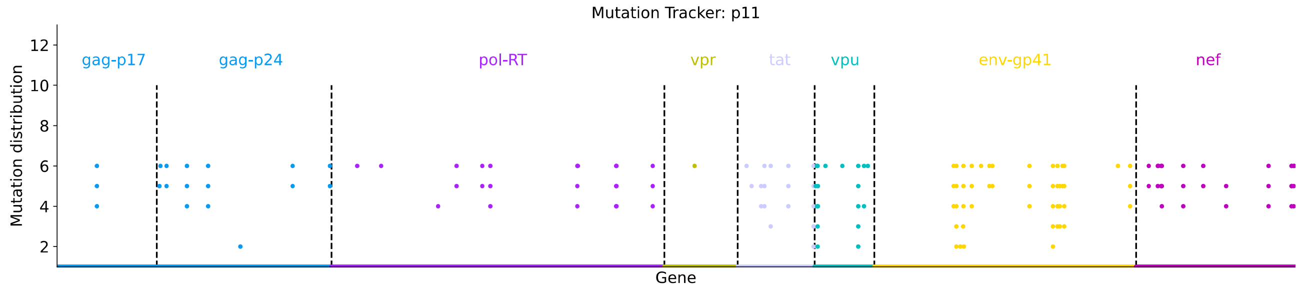

(b)

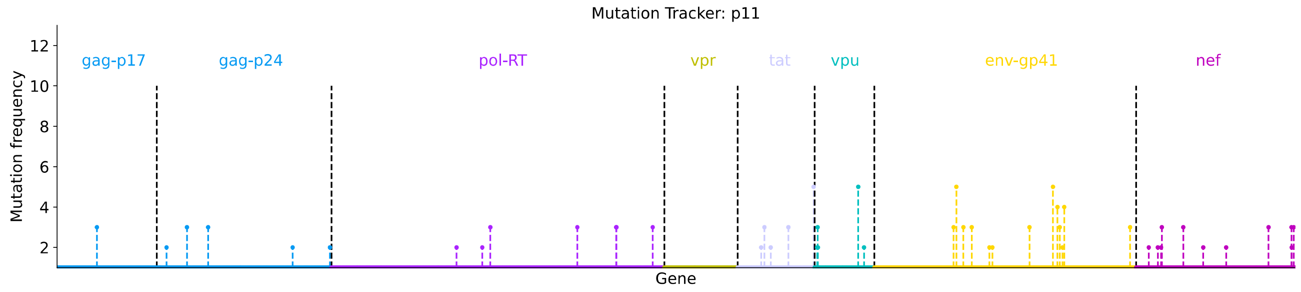

(c)

Figure S30. (a) The average nucleotide distribution difference of the eight sequence segments for patient 11 in Dataset 1 over all time periods. (b) Single nucleotide mutation distributions of the sequence segments at all time points. (c) Single nucleotide mutation frequencies of the segments at all time points. The sequence at the first time point is regarded as reference sequence.

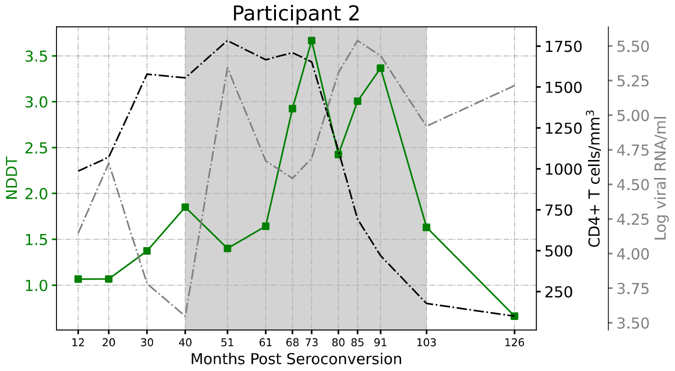

(a)

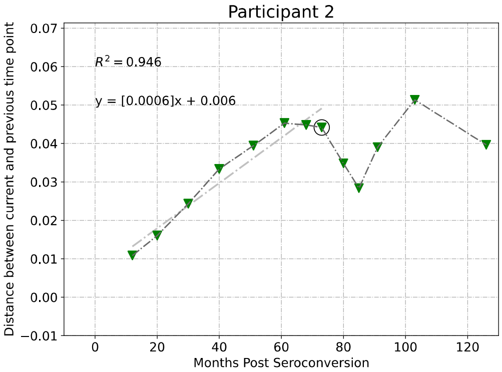

(b)

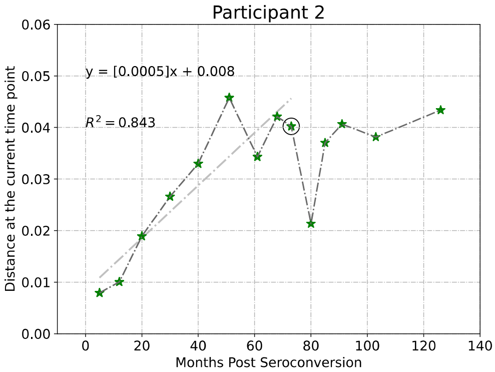

(c)

Figure S31. (a) The variation trends of C2-V5 region of HIV-1 env gene for participant 2. The mutation progressions are shown with the filled blocks connected by the colorful line. The CD4+ T cell levels are shown with the black dotted line. The viral RNA levels are with the gray dotted line. (b) Viral divergence: Distance of sequences between the current time point and its previous time point. (c) Viral diversity: Distance of sequences at the current time point. The abscissa of the circle represents the mutation rate peak of the corresponding patient.

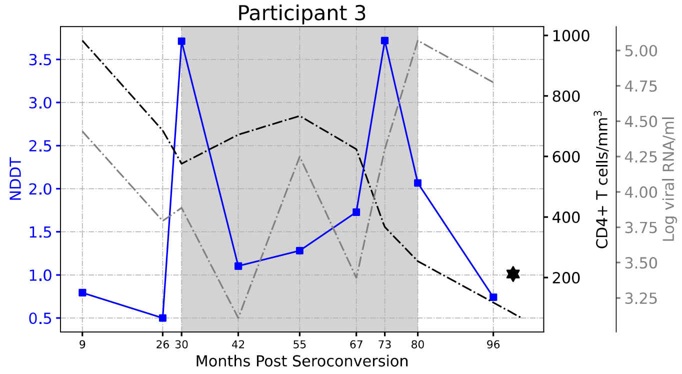

(a)

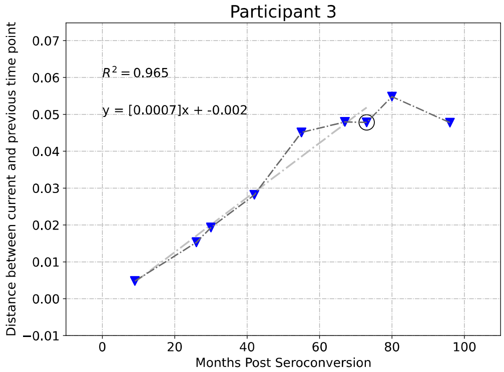

(b)

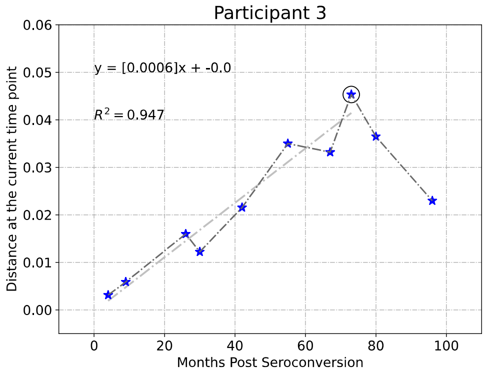

(c)

Figure S32. (a) The variation trends of C2-V5 region of HIV-1 env gene for participant 3. The mutation progressions are shown with the filled blocks connected by the colorful line. The CD4+ T cell levels are shown with the black dotted line. The viral RNA levels are with the gray dotted line. Participant 3 died after the last time point of analysis (marked with diamond ✸) (b) Viral divergence: Distance of sequences between the current time point and its previous time point. (c) Viral diversity: Distance of sequences at the current time point. The abscissa of the circle represents the mutation rate peak of the corresponding patient.

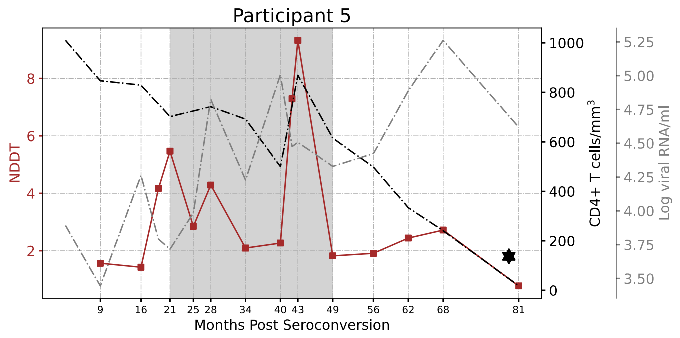

(a)

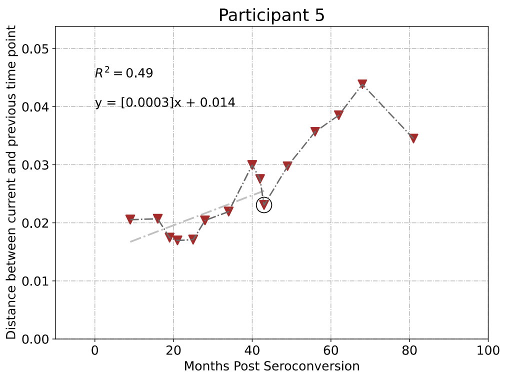

(b)

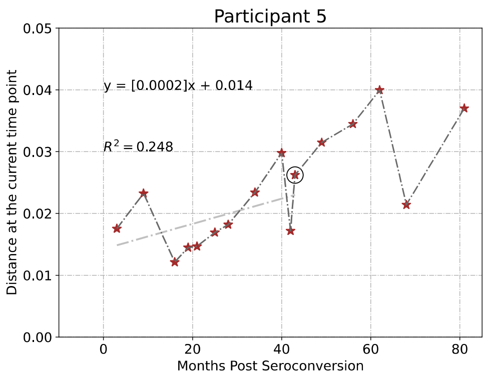

(c)

Figure S33. (a) The variation trends of C2-V5 region of HIV-1 env gene for participant 5. The mutation progressions are shown with the filled blocks connected by the colorful line. The CD4+ T cell levels are shown with the black dotted line. The viral RNA levels are with the gray dotted line. Participant 5 died after the last time point of analysis (marked with diamond ✸) (b) Viral divergence: Distance of sequences between the current time point and its previous time point. (c) Viral diversity: Distance of sequences at the current time point. The abscissa of the circle represents the mutation rate peak of the corresponding patient.

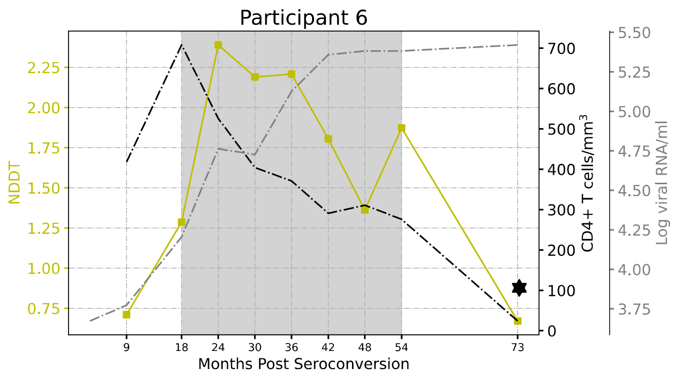

(a)

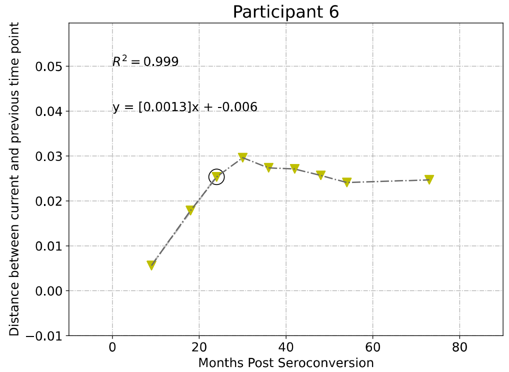

(b)

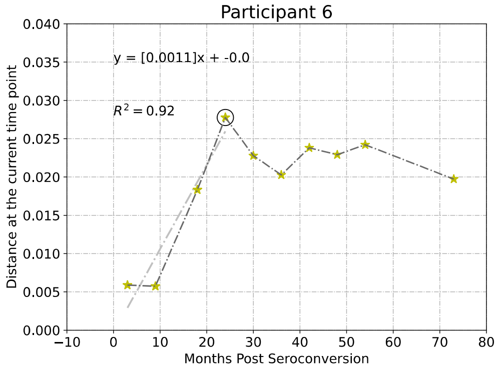

(c)

Figure S34. (a) The variation trends of C2-V5 region of HIV-1 env gene for participant 6. The mutation progressions are shown with the filled blocks connected by the colorful line. The CD4+ T cell levels are shown with the black dotted line. The viral RNA levels are with the gray dotted line. Participant 6 died after the last time point of analysis (marked with diamond ✸) (b) Viral divergence: Distance of sequences between the current time point and its previous time point. (c) Viral diversity: Distance of sequences at the current time point. The abscissa of the circle represents the mutation rate peak of the corresponding patient.

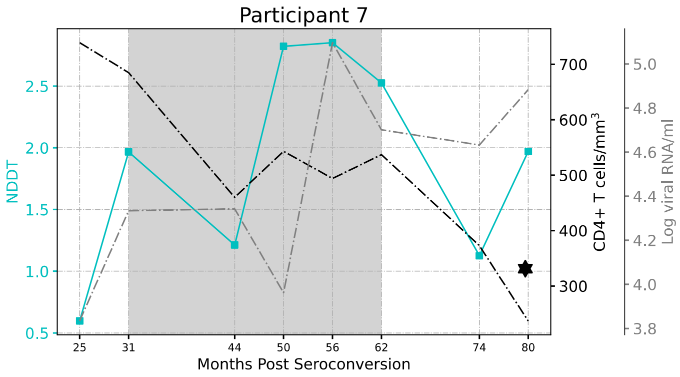

(a)

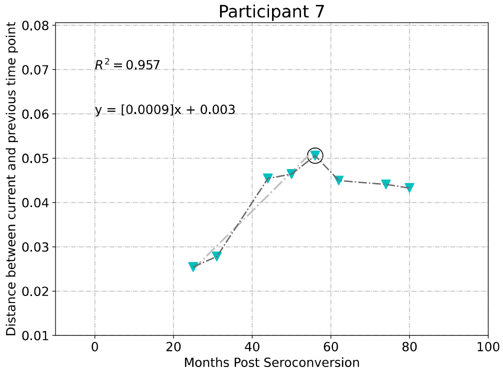

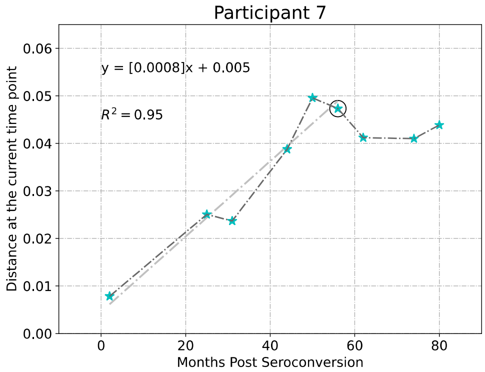

(b)

(c)

Figure S35. (a) The variation trends of C2-V5 region of HIV-1 env gene for participant 7. The mutation progressions are shown with the filled blocks connected by the colorful line. The CD4+ T cell levels are shown with the black dotted line. The viral RNA levels are with the gray dotted line. Participant 7 died after the last time point of analysis (marked with diamond ✸) (b) Viral divergence: Distance of sequences between the current time point and its previous time point. (c) Viral diversity: Distance of sequences at the current time point. The abscissa of the circle represents the mutation rate peak of the corresponding patient.

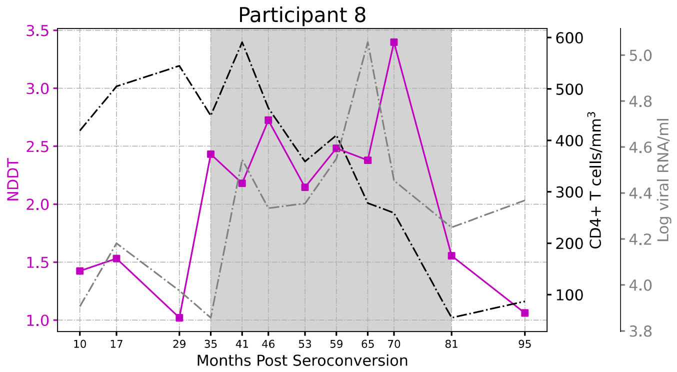

(a)

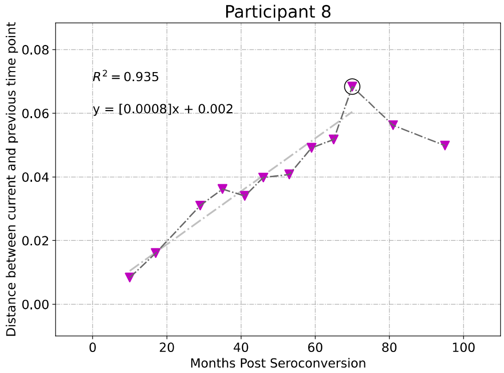

(b)

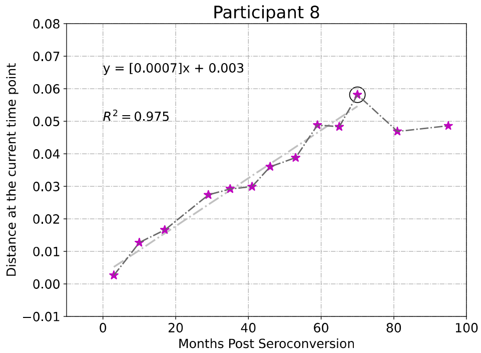

(c)

Figure S36. (a) The variation trends of C2-V5 region of HIV-1 env gene for participant 8. The mutation progressions are shown with the filled blocks connected by the colorful line. The CD4+ T cell levels are shown with the black dotted line. The viral RNA levels are with the gray dotted line. (b) Viral divergence: Distance of sequences between the current time point and its previous time point. (c) Viral diversity: Distance of sequences at the current time point. The abscissa of the circle represents the mutation rate peak of the corresponding patient.

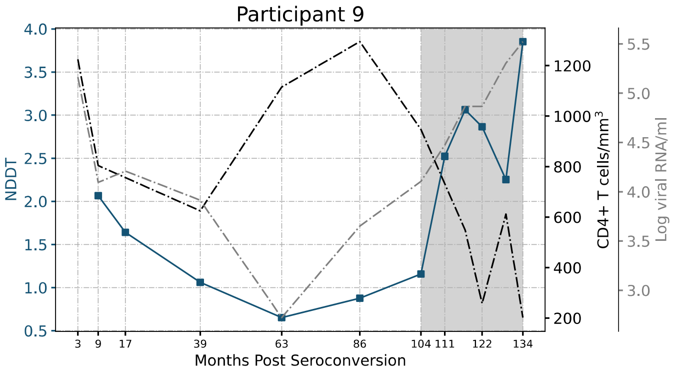

(a)

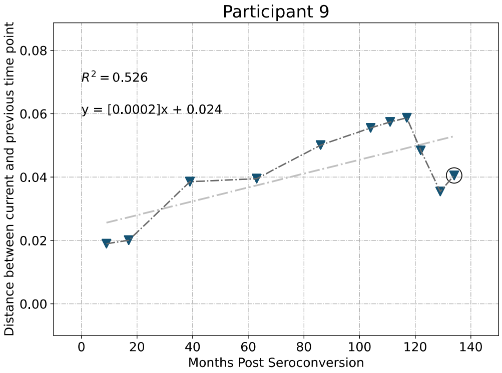

(b)

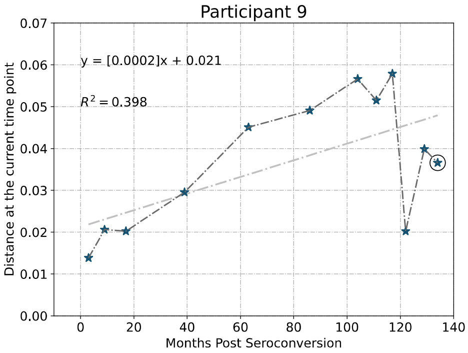

(c)

Figure S37. (a) The variation trends of C2-V5 region of HIV-1 env gene for participant 9. The mutation progressions are shown with the filled blocks connected by the colorful line. The CD4+ T cell levels are shown with the black dotted line. The viral RNA levels are with the gray dotted line. (b) Viral divergence: Distance of sequences between the current time point and its previous time point. (c) Viral diversity: Distance of sequences at the current time point. The abscissa of the circle represents the mutation rate peak of the corresponding patient.

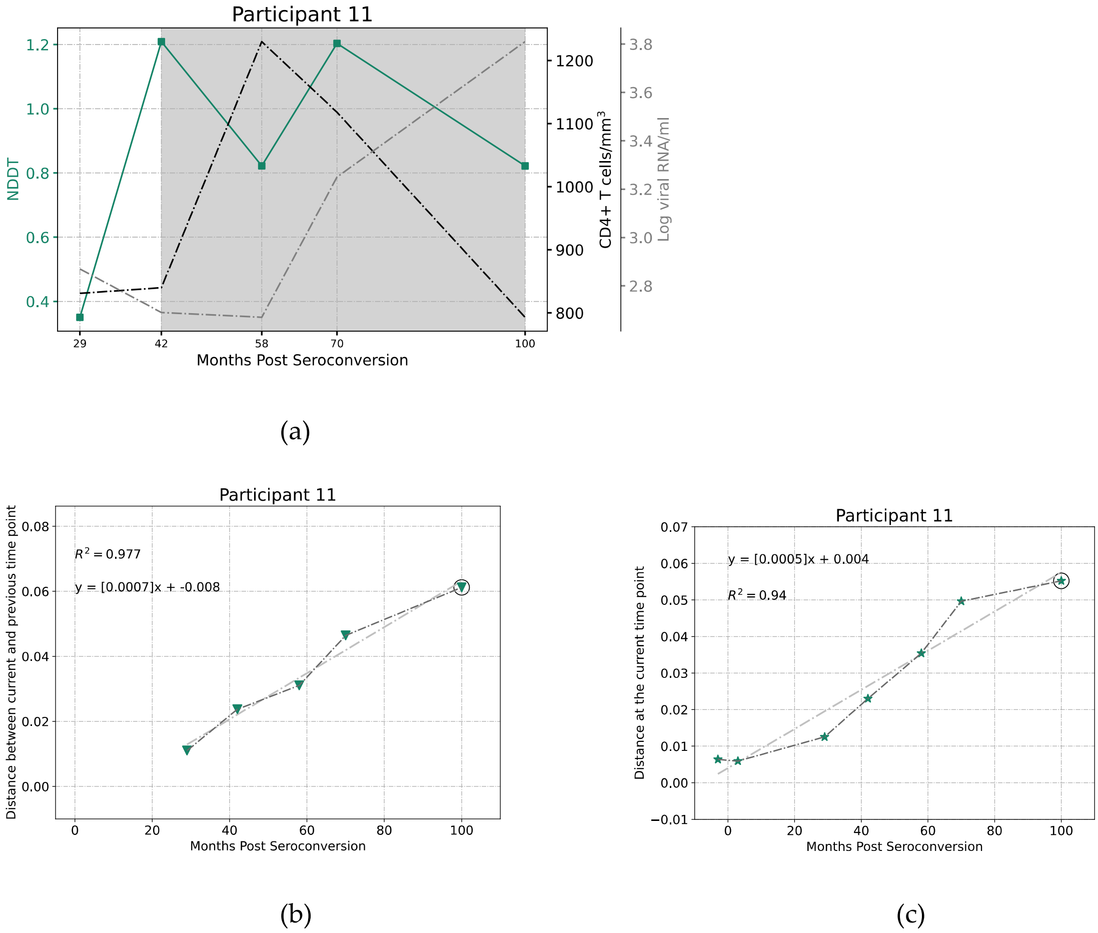

Figure S38. (a) The variation trends of C2-V5 region of HIV-1 env gene for participant 11. The mutation progressions are shown with the filled blocks connected by the colorful line. The CD4+ T cell levels are shown with the black dotted line. The viral RNA levels are with the gray dotted line. (b) Viral divergence: Distance of sequences between the current time point and its previous time point. (c) Viral diversity: Distance of sequences at the current time point. The abscissa of the circle represents the mutation rate peak of the corresponding patient.

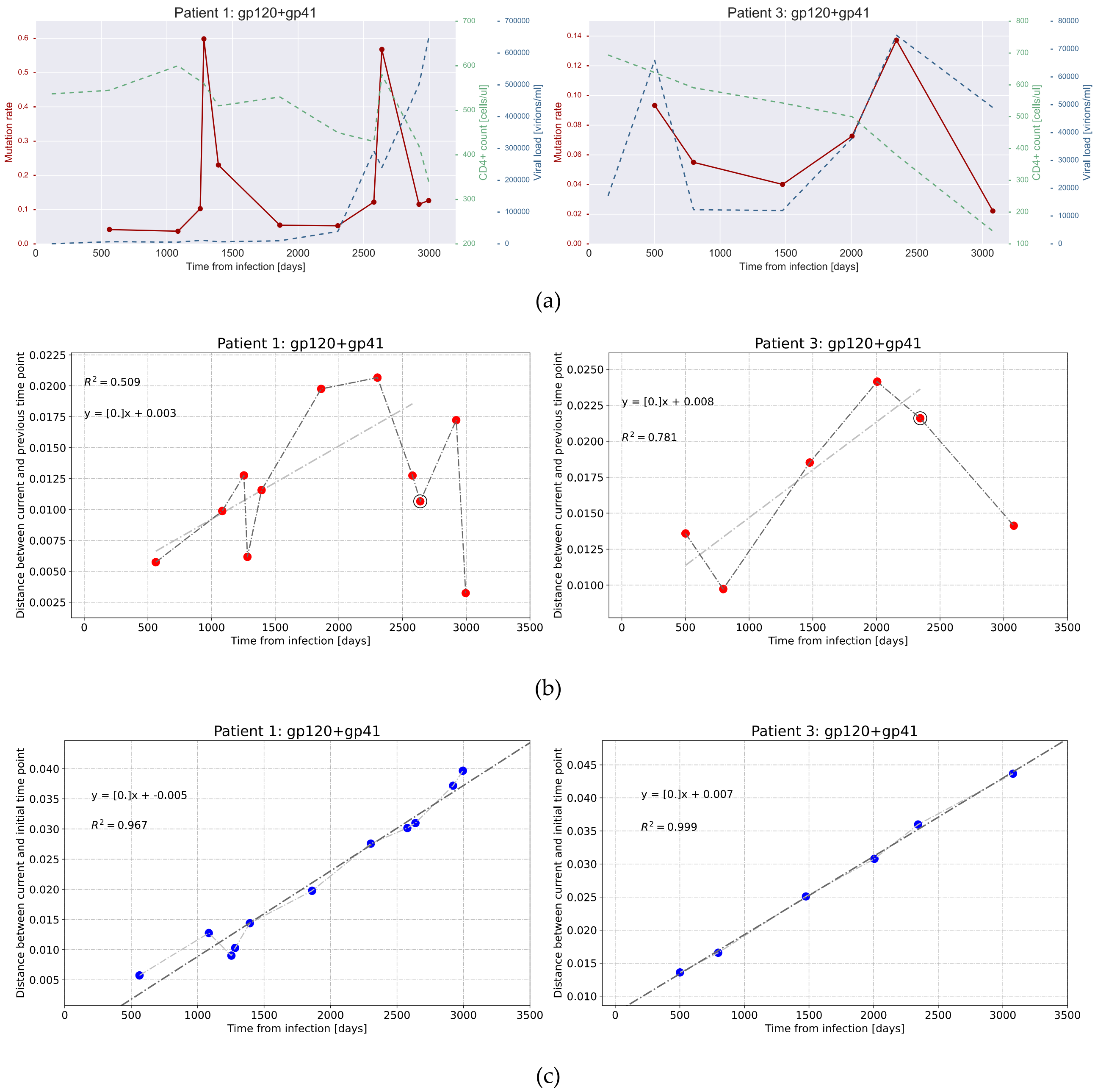

Figure S39. (a) Mutation rate of Env gene sequence. (b) Sequence distance between the current time point and its previous time point. (c) Sequence distance between the current time point and the initial time point.

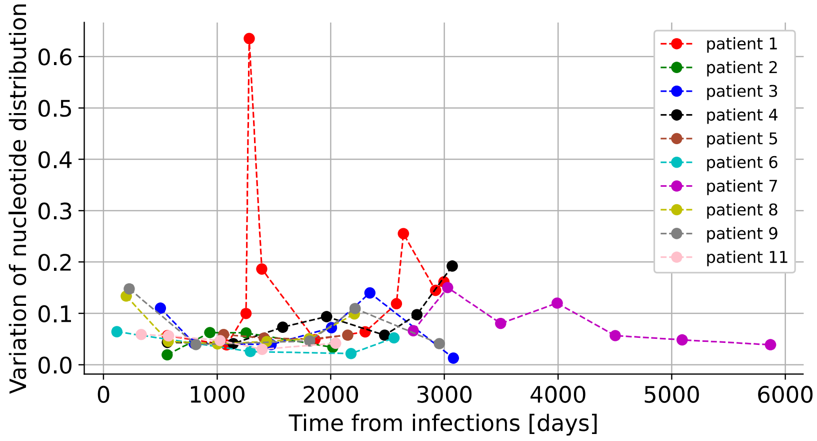

Figure S40. Mutation rate analysis of complete genomes. The analyses in Results part have demonstrated the efficiency of our NDDT definition, so we used it to analyze the mutations of complete genomes in dataset 1. The result shows that the NDDTs during all time period range from 0 to 0.2 except for patient 1.
